# Supplementary material for: Single-cell analysis reveals altered tumor microenvironments of relapse- and remission-associated pediatric acute myeloid leukemia
Source: Nat Commun. 2023 Oct 5;14:6209. doi: 10.1038/s41467-023-41994-0 (PMC10556066; doi:10.1038/s41467-023-41994-0)
Supplement: Supplementary file 1 — Supplementary Information [file 41467_2023_41994_MOESM1_ESM.pdf]

Supplementary Materials

Single cell analysis reveals altered tumor microenvironments of relapse- and remission-associated pediatric acute myeloid leukemia

Hope Mumme[1*], Beena E. Thomas[2,3*], Swati S. Bhasin[2,3], Upaasana Krishnan[1,4], Bhakti Dwivedi[5], Pruthvi Perumalla[4], Debasree Sarkar[1,3], Gulay B. Ulukaya[1], Himalee S. Sabnis[2,3], Sunita I. Park[6], Deborah DeRyckere[2,3], Sunil S. Raikar[2,3], Melinda Pauly[2,3], Ryan J. Summers[2,3], Sharon M. Castellino[2,3], Daniel S. Wechsler[2,3], Christopher C. Porter[2,3], Douglas K. Graham[2,3], Manoj Bhasin[1-4#]

1. Department of Biomedical Informatics, Emory University School of Medicine, Atlanta, GA
2. Aflac Cancer and Blood Disorders Center, Children's Healthcare of Atlanta, Atlanta, GA
3. Department of Pediatrics, Emory University School of Medicine, Atlanta, GA
4. Department of Biomedical Engineering, Georgia Institute of Technology, Atlanta, GA
5. Department of Biostatistics and Bioinformatics Shared Resource, Winship Cancer Institute, Emory University, Atlanta, GA
6. Department of Pathology, Children's Healthcare of Atlanta, Department of Pathology and Laboratory Medicine, Emory University School of Medicine, Atlanta, GA

* Equal Contribution

#***Corresponding author***:

Manoj K. Bhasin, MS, PhD.

Aflac Cancer and Blood Disorders Center

Children Healthcare of Atlanta

Health Sciences Research Building, Room N320

1760 Haygood drive

Emory School of Medicine

Atlanta, GA 30322

Emails: manoj.bhasin@emory.edu

Telephone: (404) 712-9849

Supplementary Figures

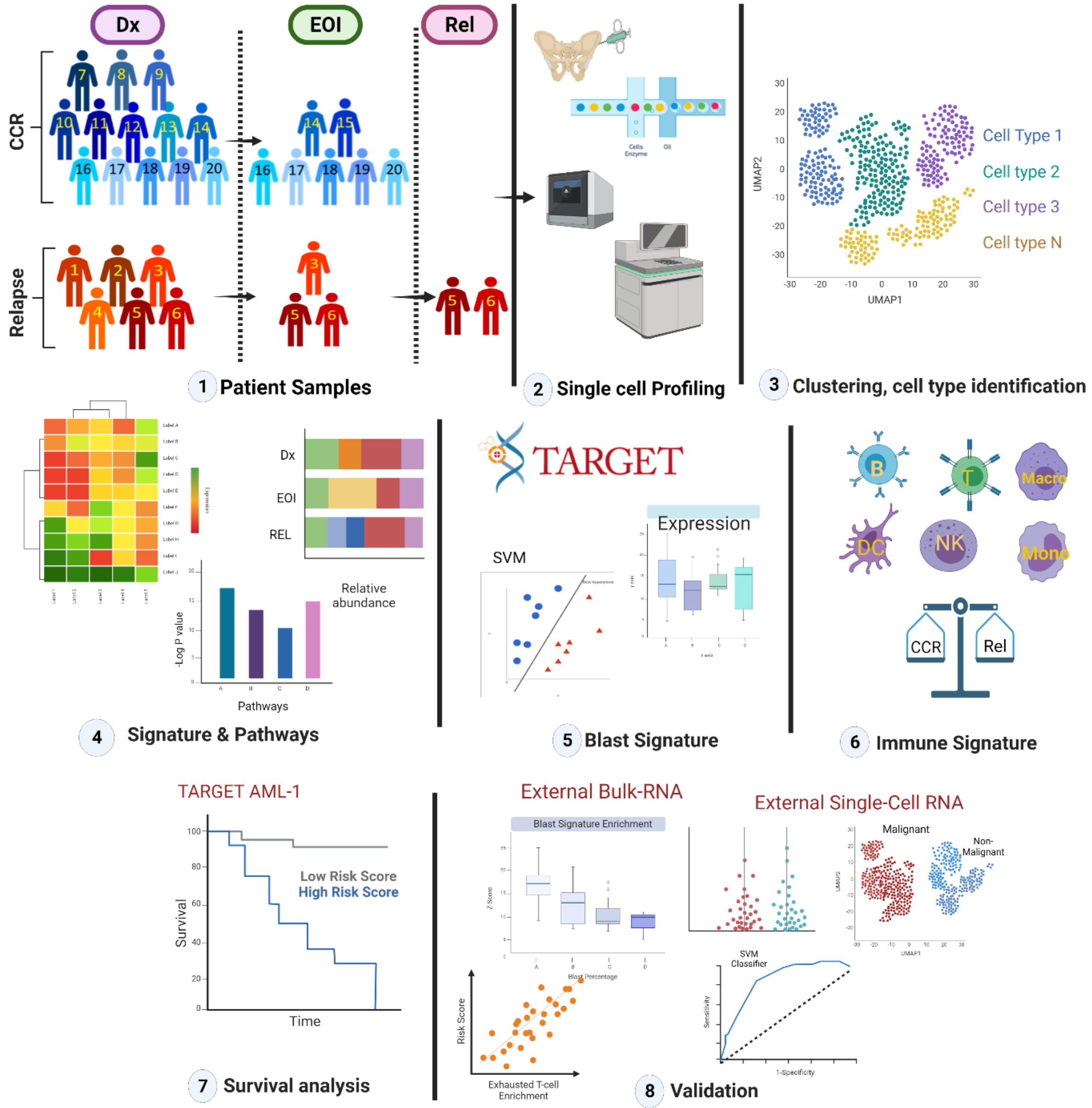

Supplementary Figure 1: Overview of study design and analytical approach. BM aspirates from AML patients with relapse (patients 1-6) and with CCR (patients 7-20) were collected at Dx, EOI, and Rel stages (patients with relapse are shown in shades of red and those with CCR are in shades of blue). ScRNA-seq libraries prepared from the viably thawed samples were sequenced and analyzed to identify blast signatures and clinical response-associated changes in cell types, transcriptomes, and pathways. The blast and immune signatures identified in the study were validated in independent single cell and bulk transcriptome studies. The schematic was created using BioRender (BioRender.com).

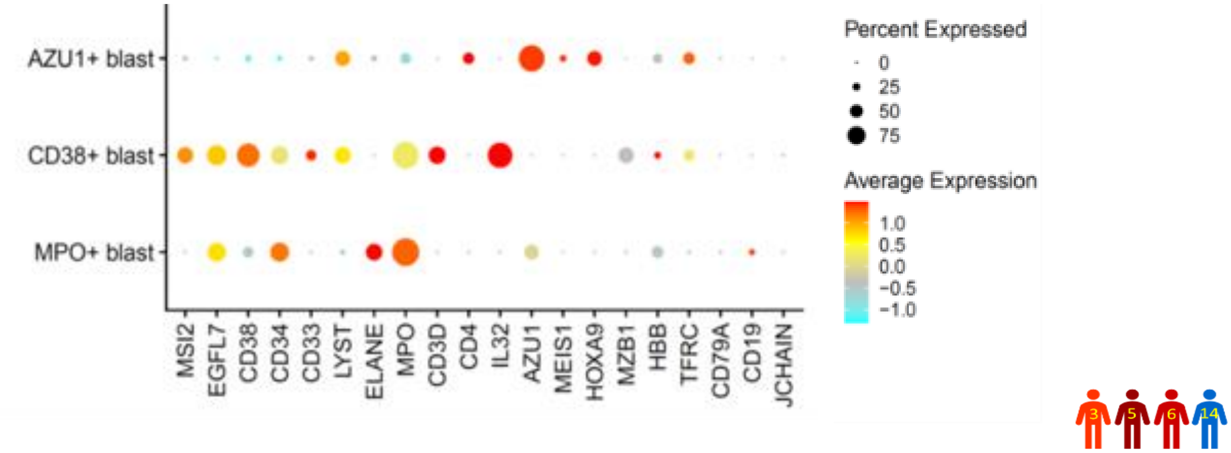

Supplementary Figure 2: Dot plot showing expression of lineage marker genes in AML-associated putative undifferentiated blast cell clusters. Dx and EOI patients' samples with relapse (3, 5, 6) and CCR (14) were analyzed. The X-axis shows genes, while the Y-axis shows the putative undifferentiated blast cell clusters. Colour scale shows gene expression levels with red, yellow and cyan representing high and low expression respectively. The size of dot represents the percentage of cells expressing each gene in individual blasts.

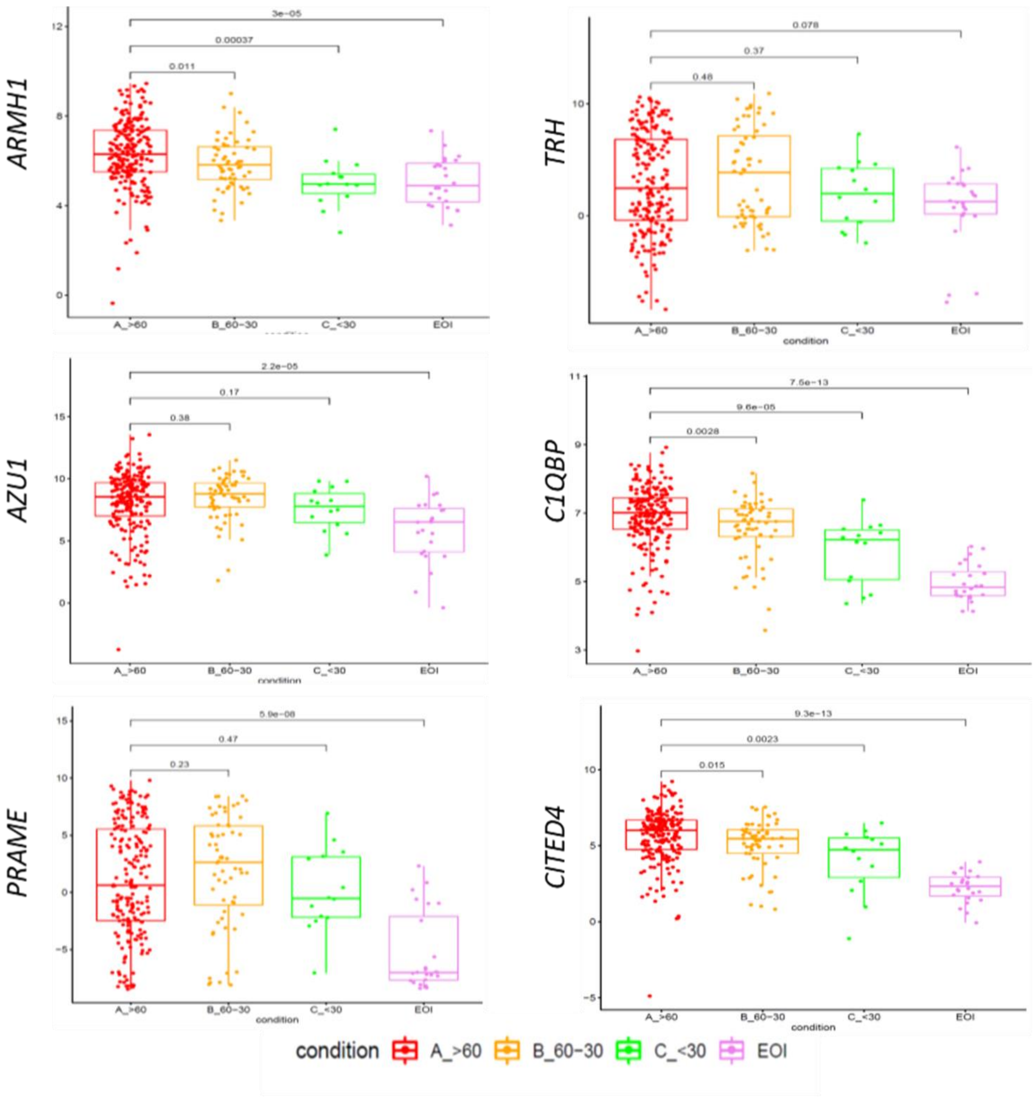

Supplementary Figure 3: Gene expression pattern of select blast-associated genes in TARGET AML-1 dataset. Box plots showing expression of select genes in AML samples collected at disease diagnosis with different percentages of blast cells (A_>60%, B_30-60%, C_<30%) and EOI samples. The X-axis is the AML categories and Y-axis is the normalized gene expression. Boxplots show the distribution of expression with the center of the box representing the median, upper and lower bounds representing 75% and 25% percentiles, and upper and lower whiskers extending to the largest value no further than 1.5 times interquartile range from bounds of box (>60% blasts group: n=203, 60-30% blasts group: n=60, <30% blasts group: n=14, EOI: n=24 biologically independent samples). Source data are provided as a Source Data file.

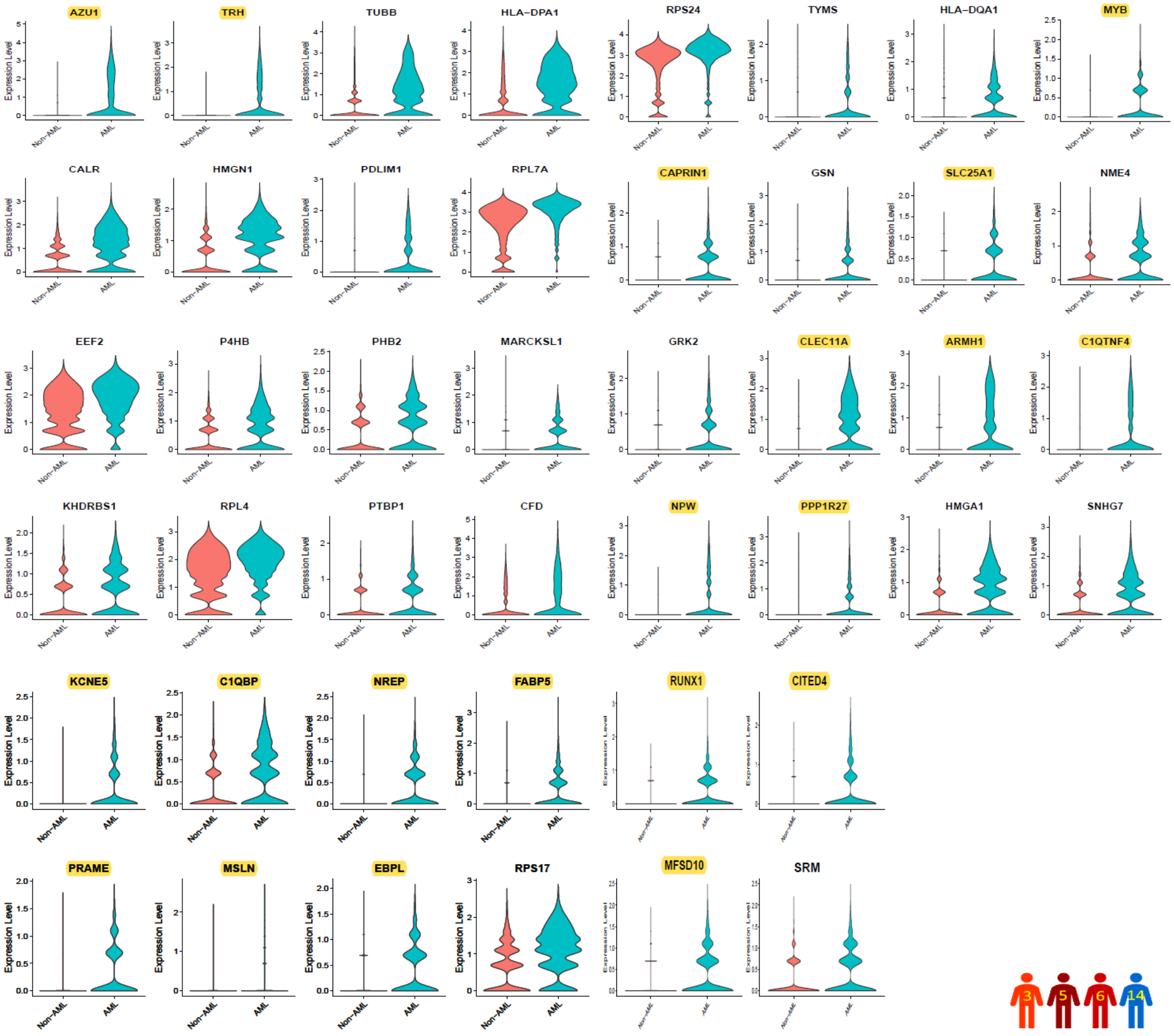

Supplementary Figure 4: Expression of twenty blast-associated genes. Expression of the 44 AML-blasts associated genes expressed highly in high blasts% TARGET AML-1 dataset samples, were analyzed in merged Dx, EOI scRNA-seq dataset (patients 3,5,6,14). The highlighted genes were the ones selected based on their specific overexpression in Dx AML-blasts and minimal expression in EOI non-blast cells. The X- and Y- axis represent the groups (AML: Dx AML-blasts; non-AML: non-blast cells), and normalized gene expression levels respectively.

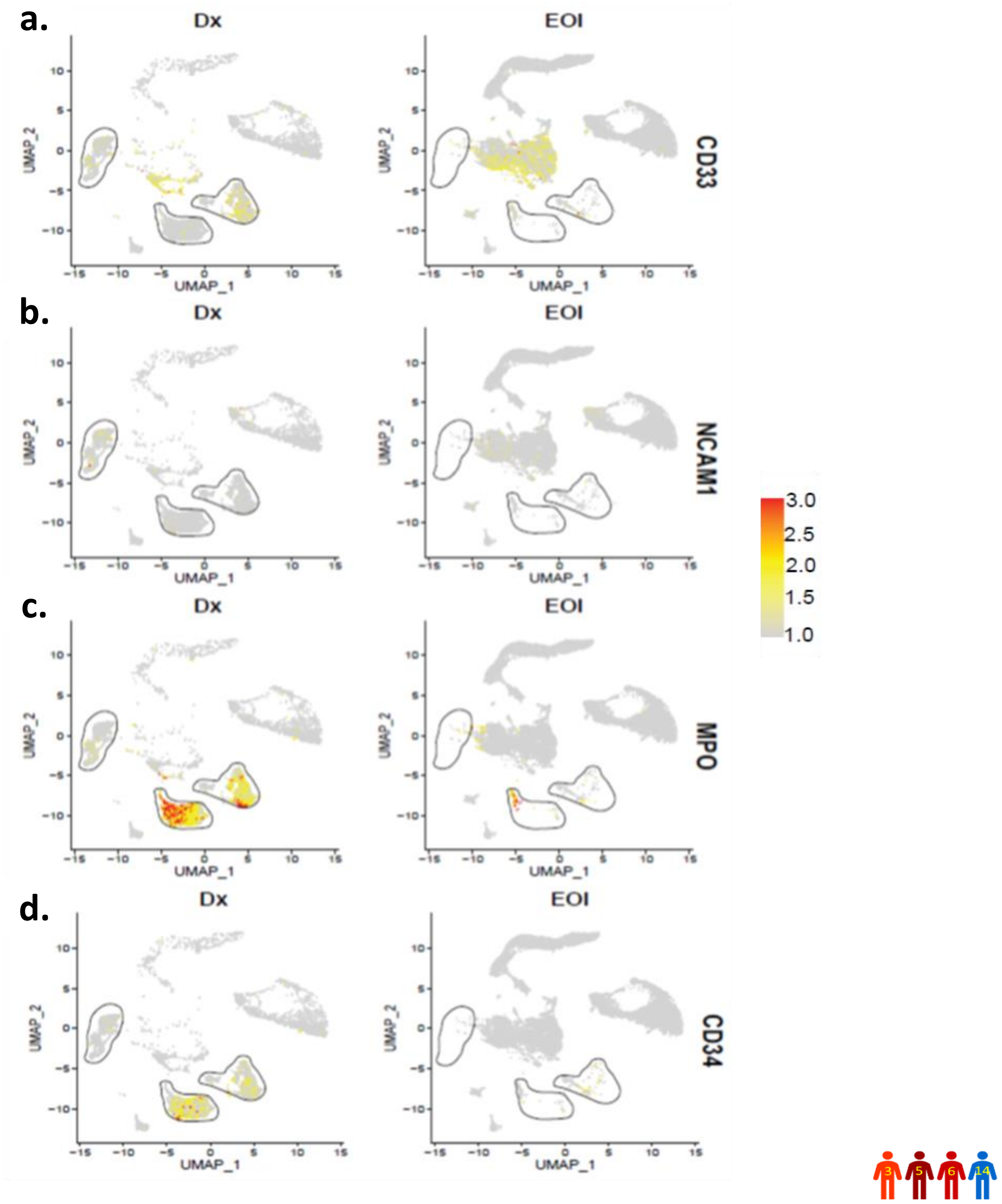

Supplementary Figure 5: Feature plots of established AML marker genes in Dx and EOI AML samples. a. *CD33*, **b.** *NCAM1/CD56,* **c.** *MPO,* **d.** *CD34* expression in the UMAPs of integrated Dx, and EOI samples from patients with relapse (3, 5, 6) and CCR (14). The putative blast clusters are lassoed. Feature plots depict normalized gene expression with red color indicating high expression, yellow, medium expression, and grey, low expression.

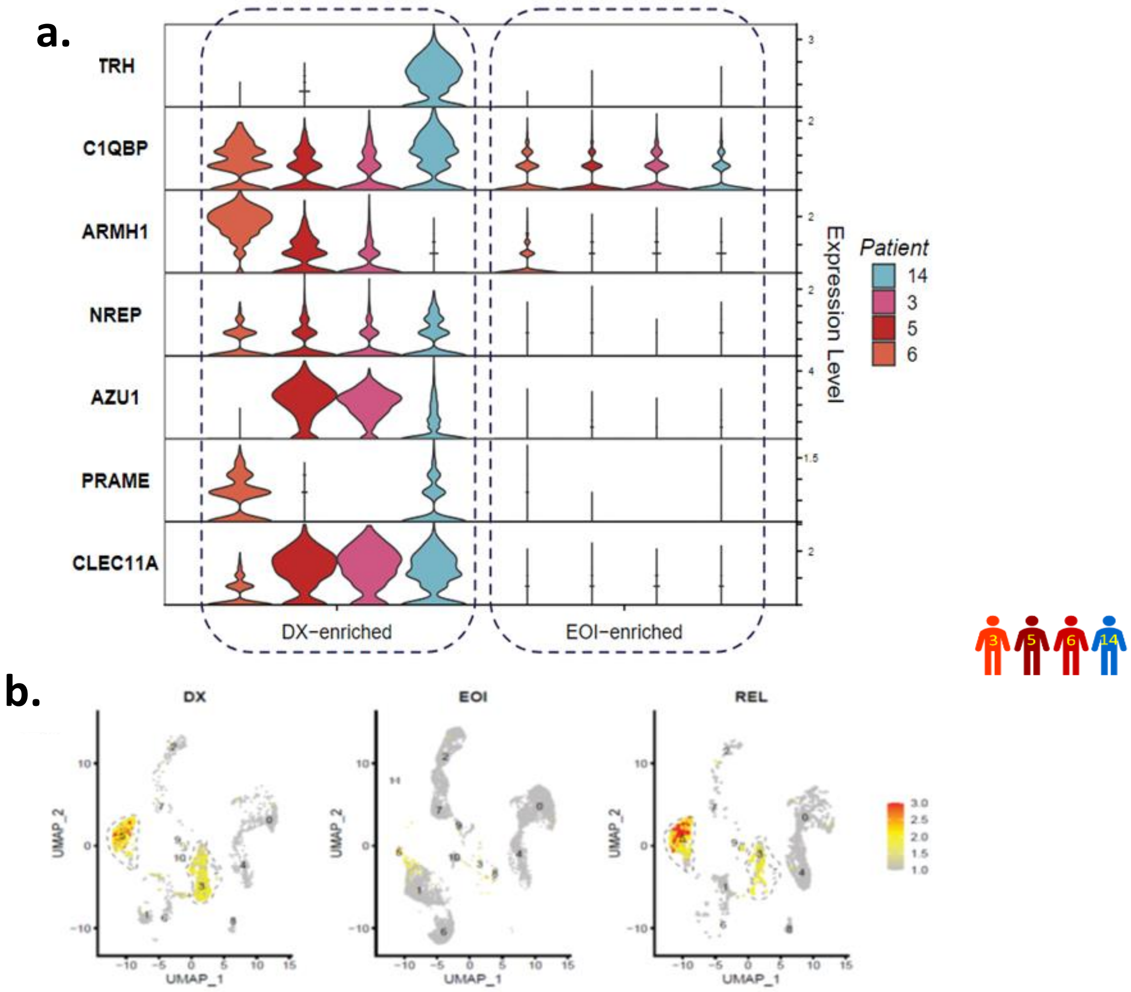

c.

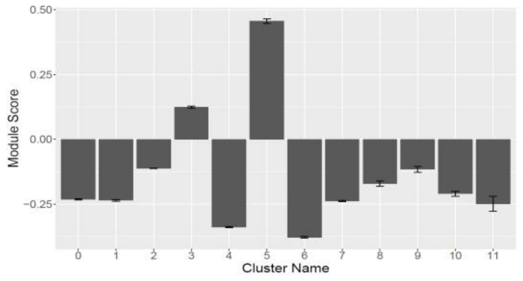

Cluster(s)	Cell type
0	T cells
1, 6	Mono/mac
3	*MPO*⁺ blast
4	T/NK cells
5	*AZU*⁺ blast
2, 7	Erythrocytes
8	B cells
9	HBB+ blast
10	pDC
11	Stromal

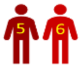

Supplementary Figure 6: AML-blast specificity of the of the 7-gene signature. a. Expression of the genes in the 7-gene signature in patients 3, 5, 6, and 14 Dx and EOI enriched clusters. X-axis represents the patient (the color key on right side indicates patient number) and Y-axis the expression of each gene from 7-gene signature. For **b, c**, samples collected at Dx, EOI, and relapse time points from two patients were analyzed**. b.** Split feature plot and **c.** Average module score plot of 7-gene signature showing increased expression of 7-gene signature in clusters 3 and 5. Module score gives us the average expression level of genes described in the module (i.e., the 7-gene signature) compared to control genes with varying expression levels. A positive/high module score in clusters 3 and 5 indicates that the gene-signature is highly expressed in these two clusters compared to the average expression of the module in the rest of the clusters. The color scale in 6**b** represents 7-gene module expression levels with red, yellow and grey representing high, medium and low expression levels.

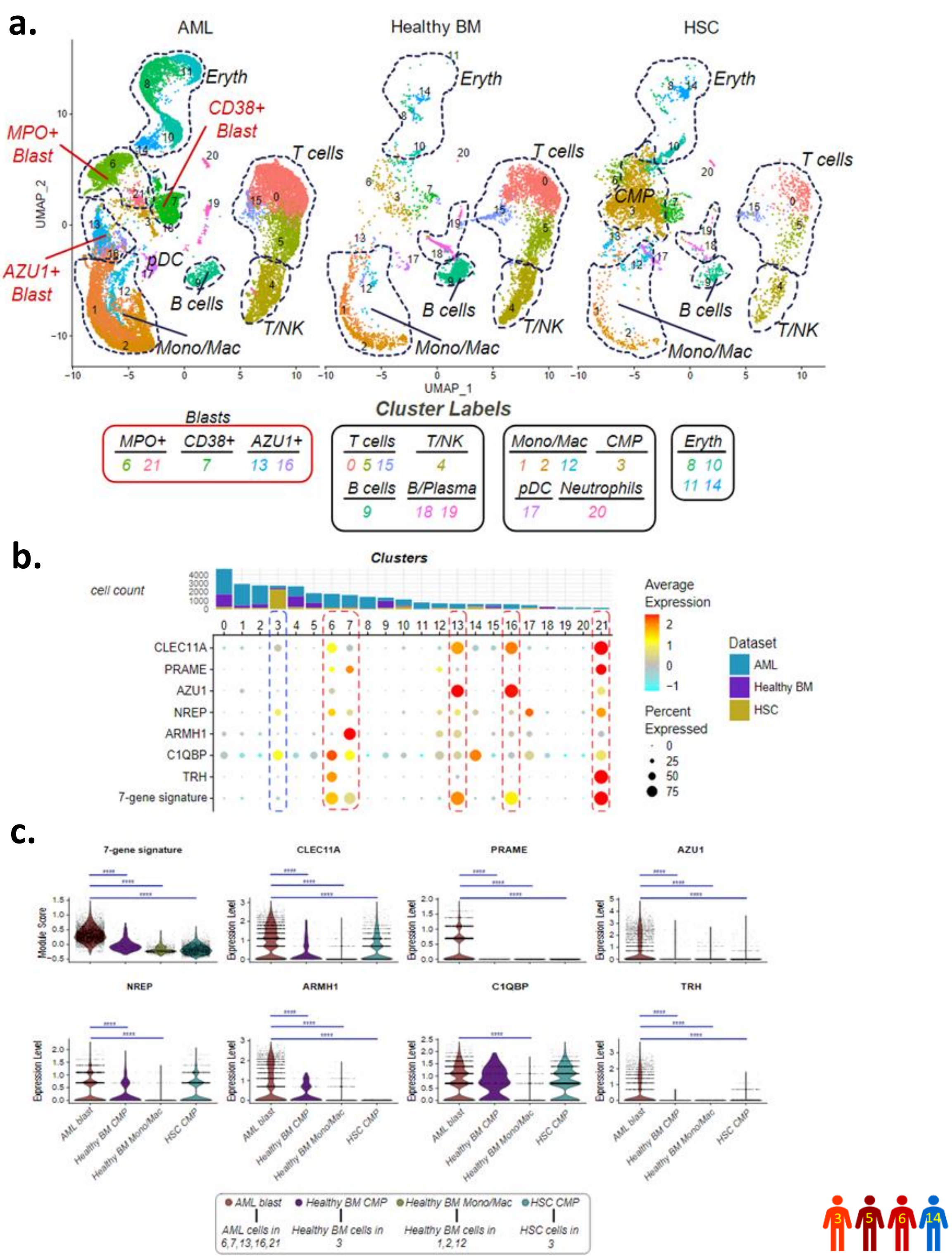

Supplementary Figure 7: Validating the AML-blast specificity of the 7-gene signature by conducting comparative gene expression analysis with healthy BMs and normal HSCs datasets. a. Split UMAP of integrated scRNA-seq data from paired Dx, EOI samples of four AML patients (3,5,6,14), healthy BMs, and normal HSCs. Large clusters are lassoed in the AML

samples, and corresponding lassoes are plotted in the Healthy BM and normal HSC clusters. Cluster labels are shown in boxes below the UMAPs. **b.** The proportion of cells in each cluster contributed from different datasets (AML, healthy BM, normal HSC) (top panel with Dataset key shown on right hand side). Dot plot with the individual genes' expression and module score of the 7-gene signature with average expression/enrichment (low: cyan, medium: grey, red: high), percent expressed (dot size) for each cluster (bottom panel). Red and blue rechanges indicate blast and **C**ommon **M**yeloid **P**rogenitor (CMP) clusters. **c.** Enhanced violin plots with the distribution of single-cell expression (log-normalized) of genes in the 7-gene signature and module score (calculated using *Seurat's* AddModuleScore function) of the signature illustrate their blast-enriched expression profile. Groups of interest are shown, AML-blasts (AML cells in clusters 6, 7, 13, 16, 21: n=4,219 cells from 4 biologically independent samples), healthy BM CMPs (healthy BM cells in cluster 3: n=164), healthy BM monocytes/macrophages (healthy BM cells in clusters 1, 2, 12: n=816), and normal HSC CMPs (HSCs in cluster 3: n=2,280). The HSC dataset was retrieved from a larger immune cell cohort from the Human Cell Atlas data portal; cells labeled as HSC were extracted to form the normal HSC dataset. The Wilcoxon rank sum test (two-tailed) was performed between AML-blasts and other groups to determine the significance of expression differences. The significant results, with AML blasts having higher expression, are shown with asterisks (**** $P<.0001$; *** $P<.001$). Source data are provided as a Source Data file.

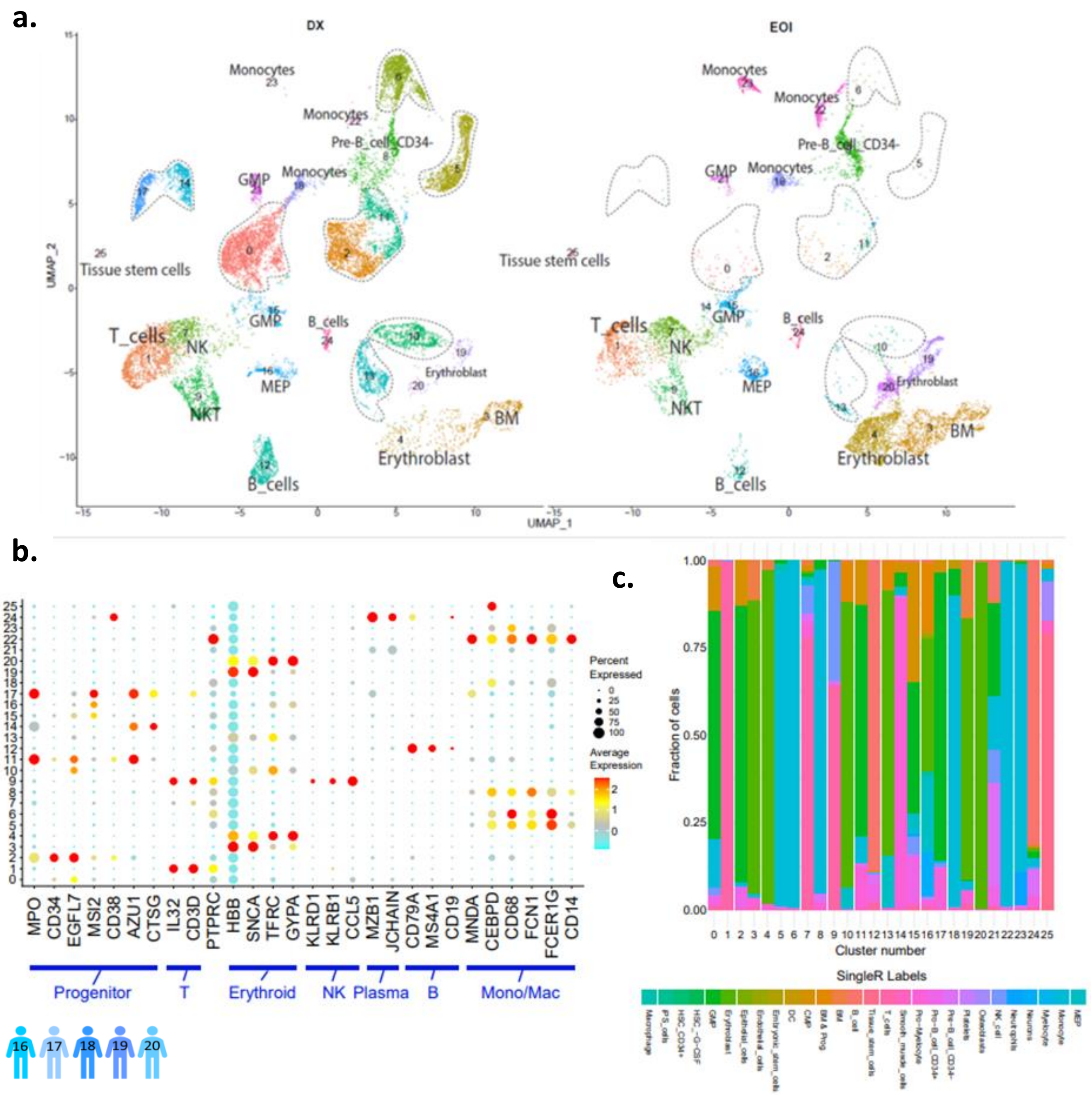

Supplementary Figure 8: Analysis of additional Dx, and EOI AML samples for independent validation of the 7-gene signature. a. To identify blast cells, we performed an integrated analysis of Dx, and EOI scRNA-seq data from patients 16-20. Clusters that are enriched at Dx and reduced at EOI (0,2,5,6,10,11,13,14,17) are lassoed. The non-blast cells in the UMAP have been labeled using singleR annotation tool[1]. **b.** Canonical marker expression for lymphoid, myeloid and erythroid lineage cell types in each of the clusters. The size of dots indicates the percentage of cells in each cell cluster (Y-axis) expressing the marker gene (X-axis); color represents averaged scaled expression levels; cyan: low, yellow: medium and red: high. **c.** Cluster-wise makeup of SingleR predicted cell type labels. Early myeloid (CMP, GMP, and MEP) labeled cells are concentrated in assigned blast clusters (0,2,5,6,10,11,13,14,17).

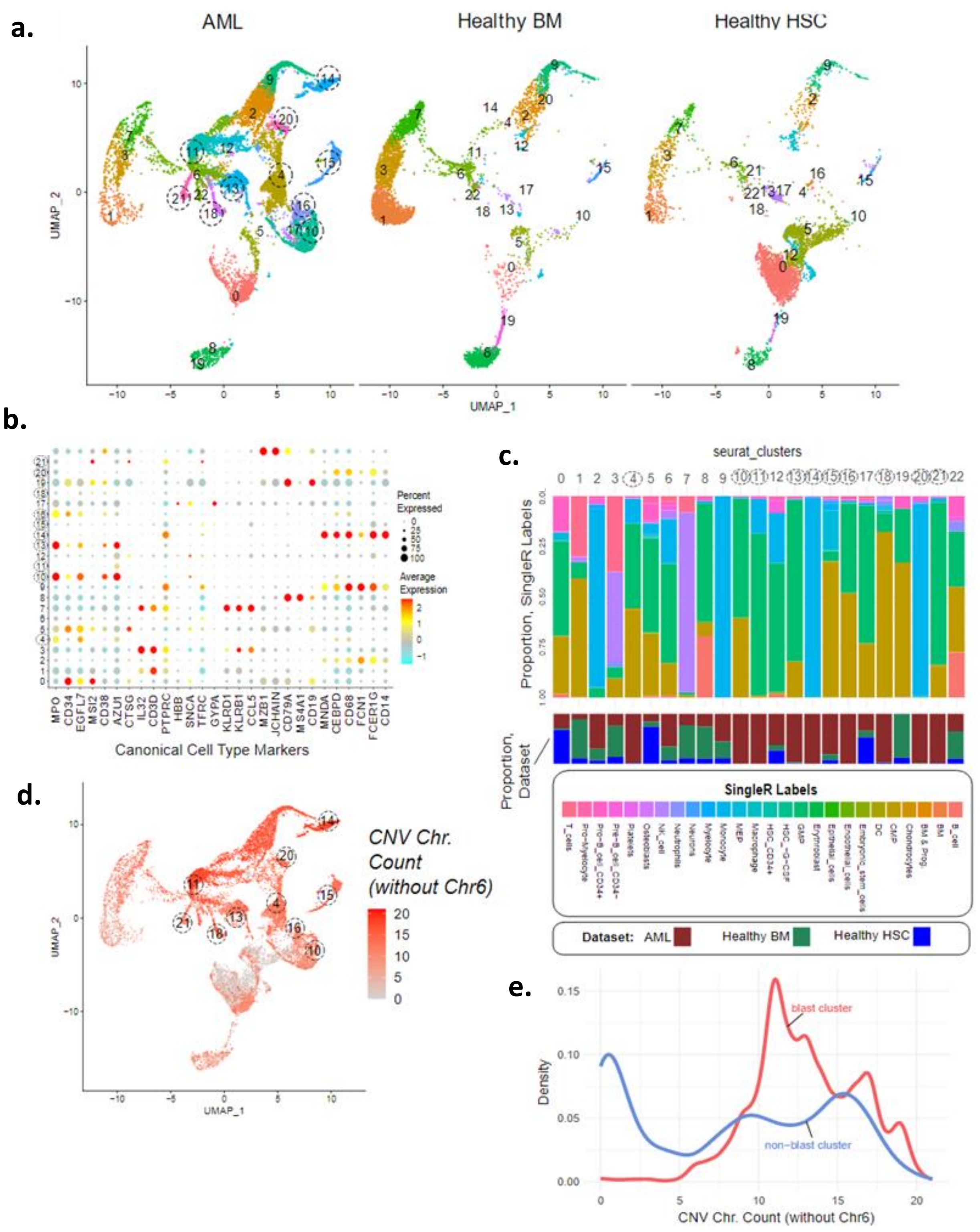

Supplementary Figure 9: Identification of blasts and non-blasts in external AML single cell dataset used for validating 7-gene signature. a. Split UMAP showing transcriptomically distinct clusters distribution in the three clinical datasets, i.e., the external validation pediatric AML (n=8)[2], young adult healthy BMs, and adult normal HSCs samples. AML-blast clusters are circled. **b.** Dot

plot showing canonical cell type markers used for annotation of clusters. Size of dots indicates percentage of cells in each cell cluster expressing the marker gene; color represents average scaled expression levels; cyan: low, yellow: medium, and red: high. **c.** Bar plots showing the proportion of *SingleR* automatic annotation cell types in each cluster (top panel). The proportion of cells in each cluster contributed from different datasets (AML, healthy BM, healthy HSC) (bottom panel). Based on annotation and low proportion in healthy and HSCs datasets, clusters were annotated as blast cells (dashed circles). **d.** CNA analysis was performed using *inferCNV*; HSC data were used as the “reference”, and AML data were input as “observations”. The CNA chromosome count represents the number of chromosomes with a predicted CNA present for each cell, not including chromosome 6. This metric is shown for each cell on the feature plot, with red representing a high count and light grey representing a low count. **e.** Density plot of CNA chromosome count distribution for AML-blast (4, 10, 11, 13-16, 18, 20, 21) and non-blast clusters shown by red and blue line respectively .

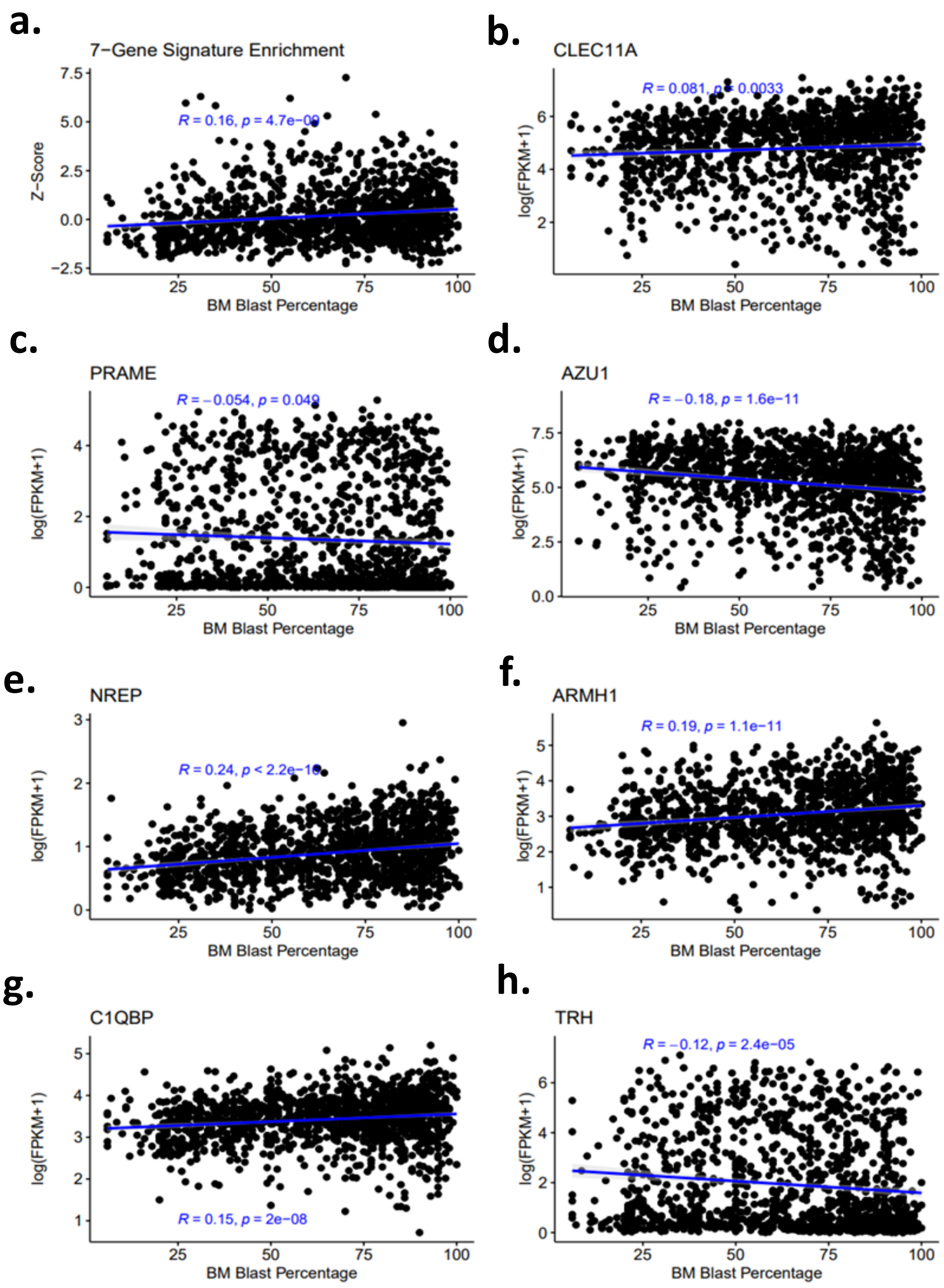

Supplementary Figure 10. Validation of 7-gene signature in additional TARGET AML samples. a. Z-scores were calculated (R package GSVA v3.17) to represent the combined expression of the 7-gene signature in the additional TARGET primary AML biologically independent samples with >5% bone marrow (BM) blast cells (TARGET AML-2, n=1,320 biologically independent samples). Scatter plot showing relative Z-scores of samples along with BM blast percentages. **b-h.** Scatter plots for individual genes in the 7-gene signature log FPKM+1 expression against BM blast percentages. Pearson's correlation tests were performed to assess the correlation of expression/enrichment of the 7-gene signature and BM blast percentage, and corresponding *P*-values are shown. Source data are provided as a Source Data file.

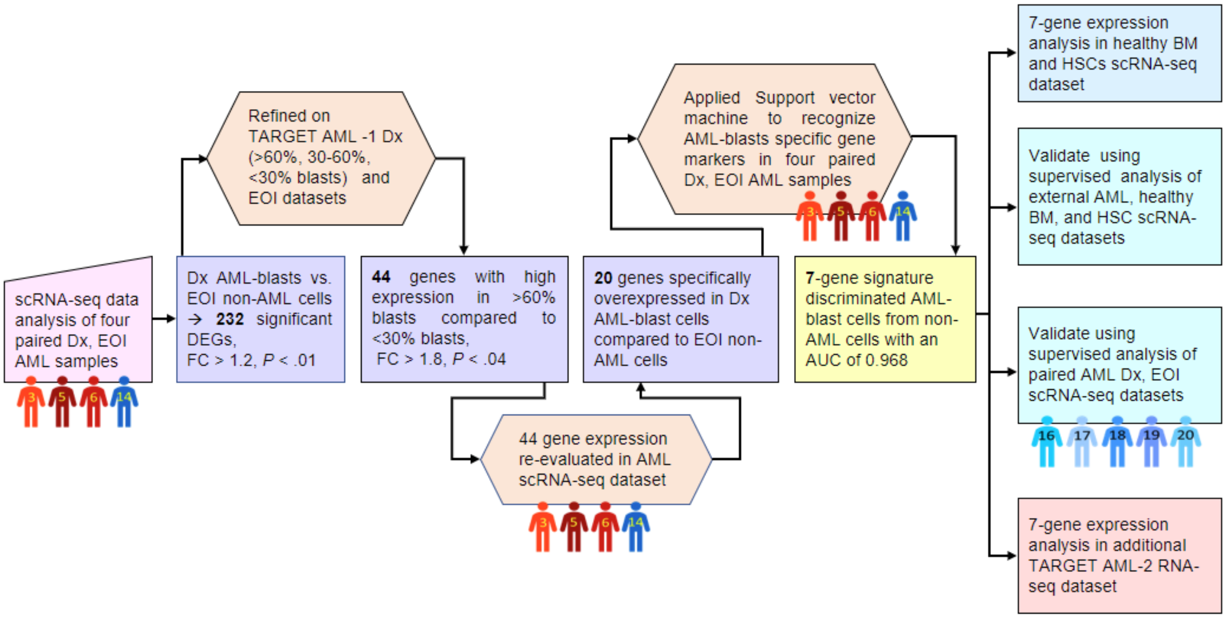

Supplementary Figure 11: Workflow utilized to develop the 7-gene AML-blast cell signature. ScRNA-seq data from four biologically independent paired Dx, EOI samples (patients 3, 5, 6, 14) were used along with TARGET AML-1 RNA-seq dataset to develop the signature. The 7-gene signature was then subjected to validation by checking expression in healthy BM samples[2], and normal HSCs (https://data.humancellatlas.org/explore/projects/cc95ff89-2e68-4a08-a234-480eca21ce79). Validation of the 7-gene signature was also carried out on two sets of AML samples scRNA-seq datasets: internal patients 16 - 20 scRNA-seq dataset and external publicly available pediatric AML scRNA-seq dataset[2]. We also evaluated the expression of 7-gene signature in bulk RNA-seq dataset of pediatric AML Dx samples (TARGET AML-2) (n=1,320 biologically independent) and its correlation with their clinical blast percentages.

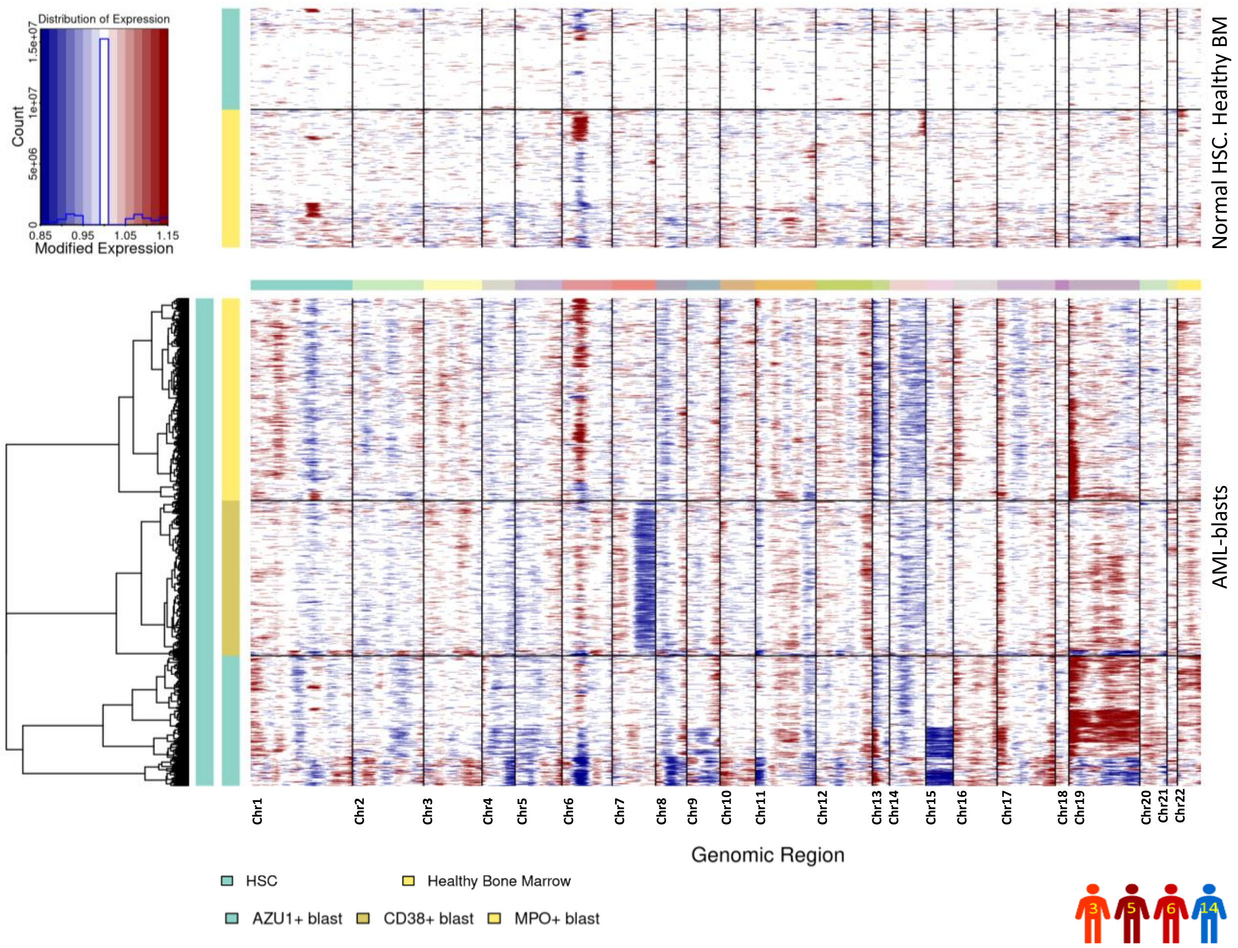

Supplementary Figure 12: Chromosome number alteration (CNA) analysis of AML-blast cells. The AML-blasts from patients 3,5,6,14 were analyzed using the inferCNV tool[3] which explores the expression intensity of genes across positions of the genome in observational cells i.e., AML-blasts (bottom panel) in comparison to a set of reference cells i.e., normal HSC and healthy BM controls (top panel). Chromosomal amplification (red) and deletion (blue) inferred across each chromosome (bars on top of the second heatmap) are represented. The predicted CNAs of AML-blasts and normal HSC, and healthy BM are shown in bottom and top heatmap, respectively. Rows in the heatmap correspond to individual cells across the three blast populations (bars on the left; *AZU+*: patients 3, 5, *CD38+*: patient 6, *MPO+*: patient 14) and columns correspond to genes ordered by chromosomal position.

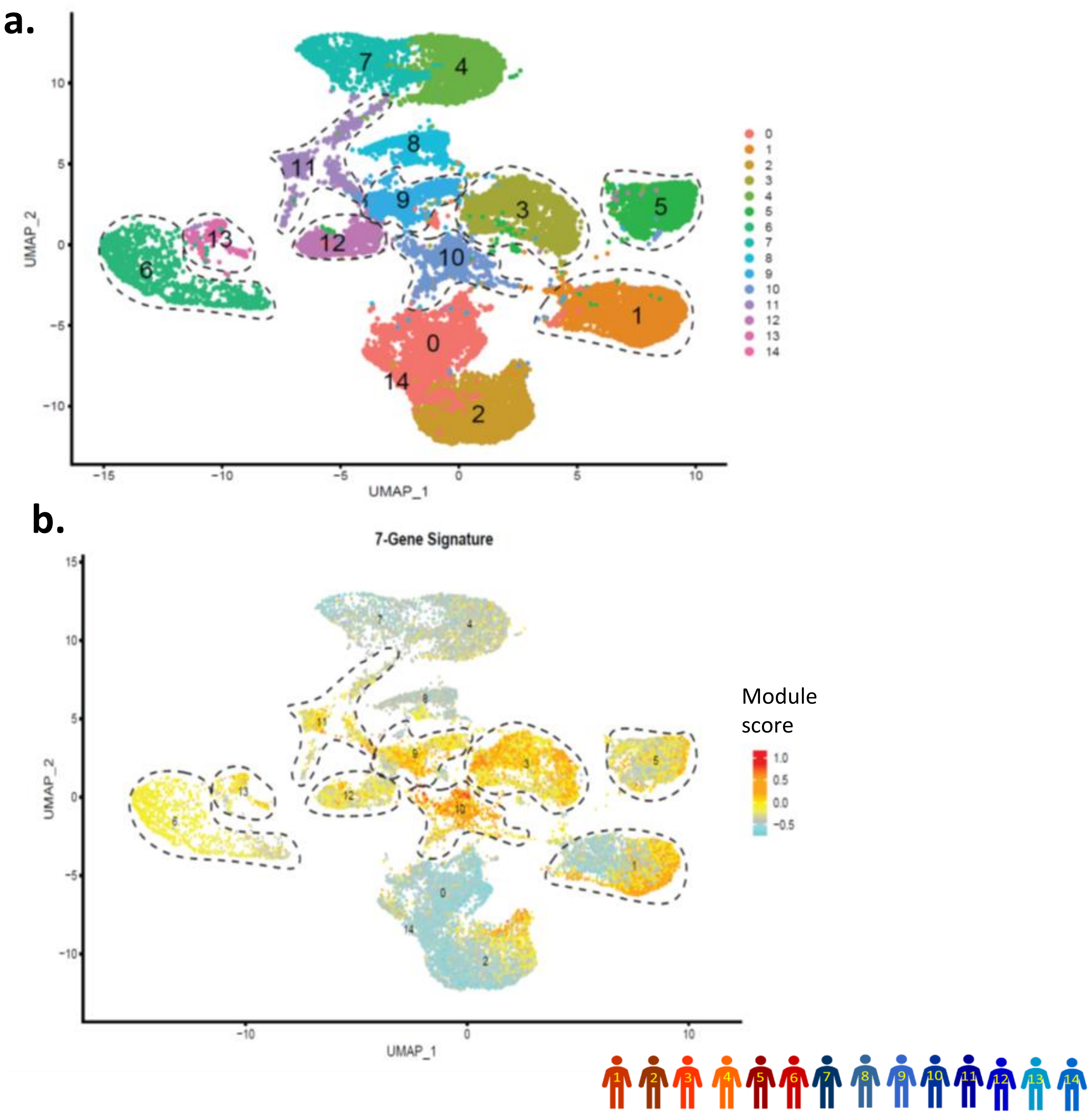

Supplementary Figure 13: Single-cell profiles of AML-blasts and non-blasts cells in Dx samples. In this analysis, Dx samples from patients with relapse (1D - 6D) and with CCR (7D - 14D) were analyzed. **a.** UMAP shows fifteen clusters colored based on transcriptome profile. **b.** Feature plot of module score based on 7-gene signature across single-cell clusters. Color scale represents module score with red and cyan representing positive and negative module scores, respectively. The blasts clusters have been lassoed.

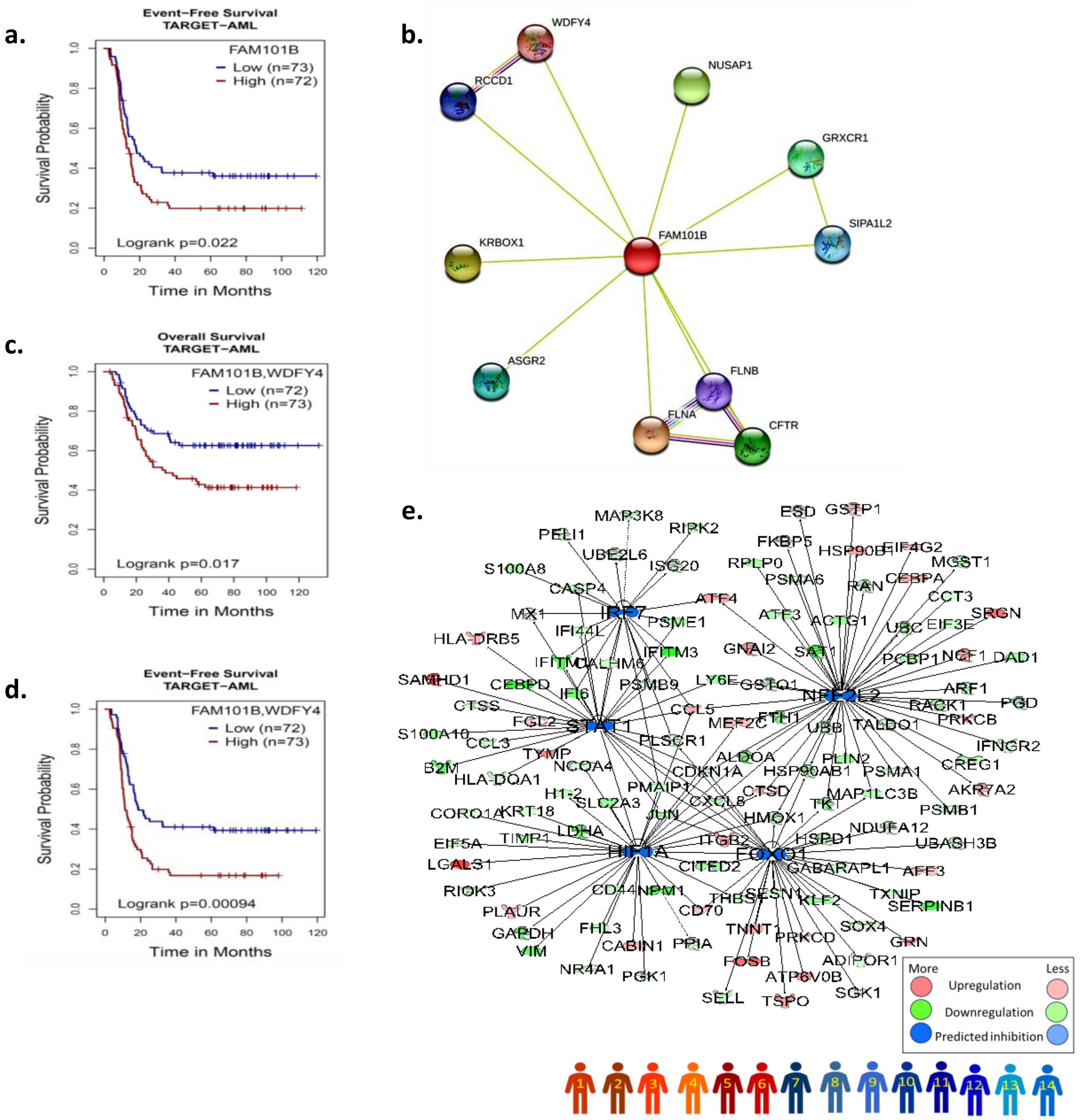

Supplementary Figure 14: Survival, network analysis of *RFLNB* gene in relapse-associated blast cells. Dx samples from patients with relapse (1D - 6D) and with CCR (7D - 14D) were analyzed. **a.** Survival genie (bhasinlab.bmi.emory.edu/SurvivalGenie/)[4] analysis revealed that higher expression of *RFLNB/FAM101B* is associated with poorer EFS in TARGET AML data. **b.** String network[5] analysis showing association of *RFLNB/FAM101B* with other genes (green lines: associations identified by text mining), **c.** Overall and **d.** Event-free survival based on co-expression of *RFLNB/FAM101B* and *WDFY4* (see also Supplementary Table 8). **e.** Upstream regulatory molecules significantly inhibited (blue) in select relapse-enriched blast clusters as compared to select CCR-enriched blast clusters.

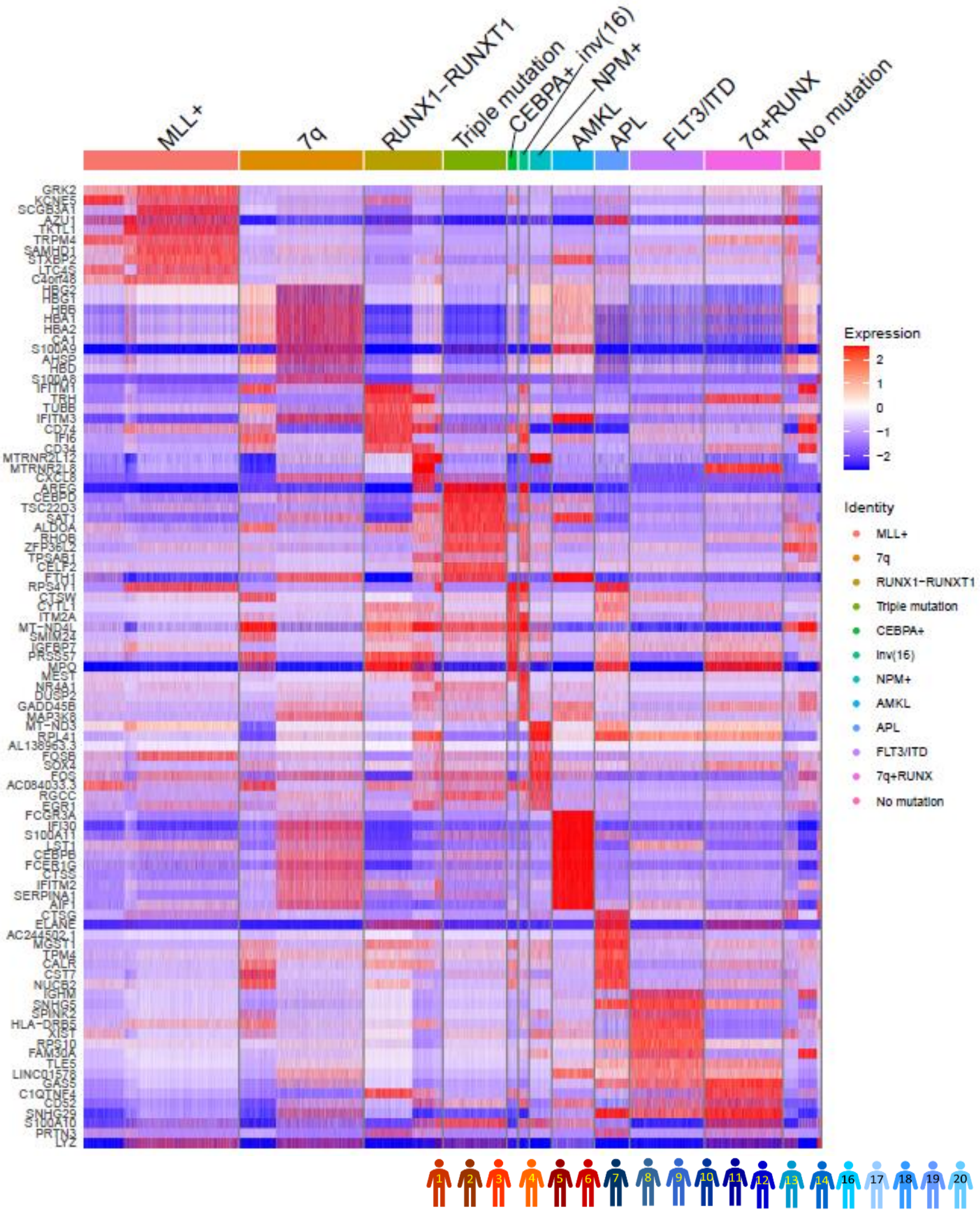

Supplementary Figure 15: DEGs in AML subtypes from individual patients. Heatmap of Dx samples (patients 1D-14D, 16D-20D) showing top DEGs among different AML subtypes. Color bars on top of heatmap show subtypes; MLL+ (patients 1,4,5), RUNX+ (patients 11,12,14,20), 7q (patient 6,16), NPM+ (patient 10) triple mutation (patient 13), CEBPA+ (patient 8), INV(16) (patient 9), no mutations (patients 2,3,7), AMKL (patient 17), APL (patient 18), FLT3/ITD (patient 19), and 7q+RUNX (patient 20). Rows represent relative gene expression with blue and red colors representing low and high expression of genes, respectively.

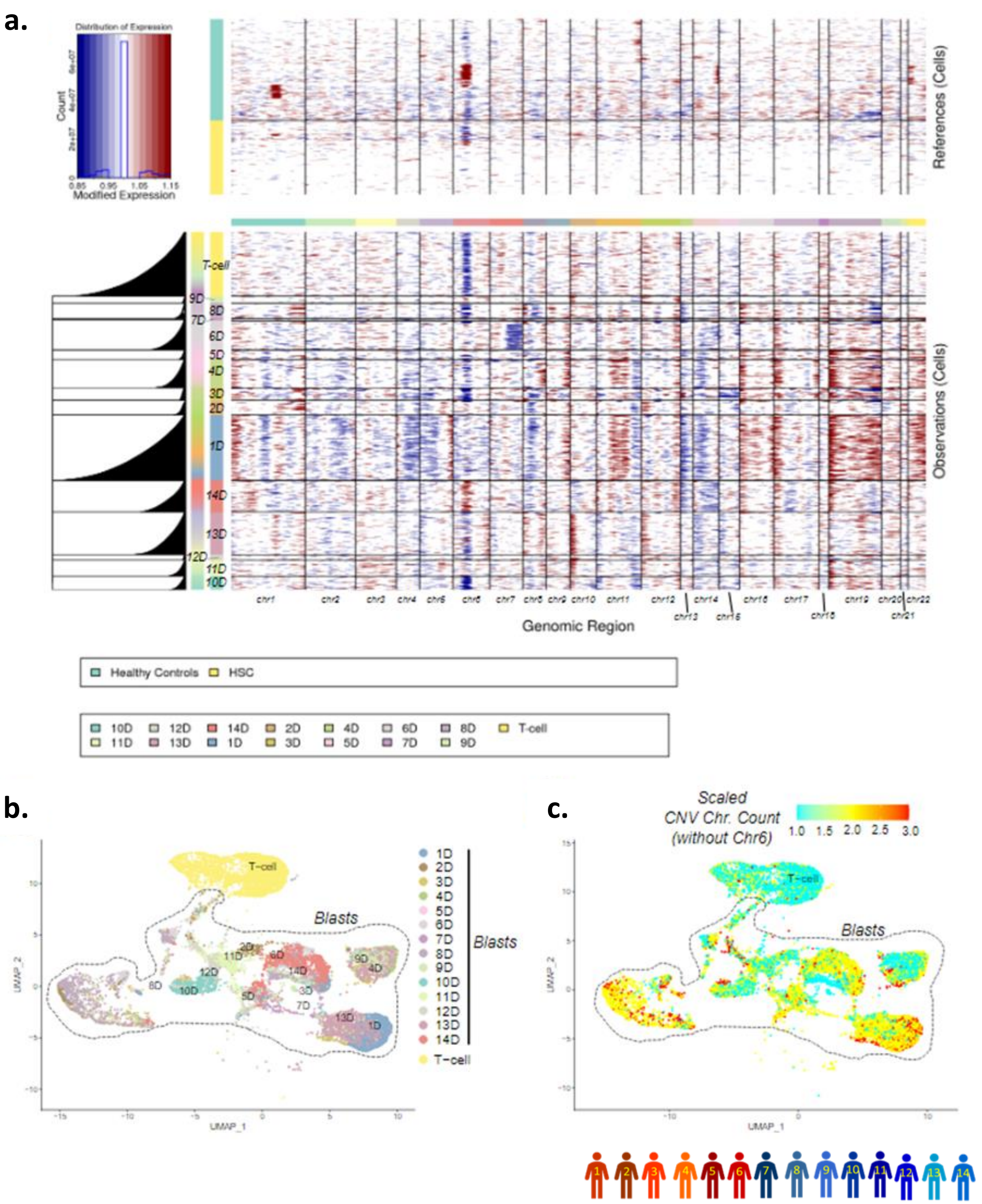

Supplementary Figure 16: CNA analysis of Dx AML-blasts cells in patient samples collected at diagnosis. a. Dx AML-blasts from patients 1-14 were analyzed using inferCNV tool[3]. The expression intensity of genes based on genomic locations in observational cells i.e., AML-blasts and AML T-cells (bottom panel), in comparison to reference cells i.e., normal HSC and healthy BM controls (top panel) was tested. Chromosomal amplification (red) and deletion (blue) inferred across each chromosome (bars on top of the second heatmap) are represented. Rows

in the heatmaps correspond to individual blast cell populations (bars on the left) and columns correspond to genes ordered by chromosomal position. **b.** UMAP shows the location of AML blast cells (colored by patient sample), and AML T-cells for the Figure S13 data. Blast cells are lassoed. **c.** Feature map of cells colored by the scaled CNA chromosome count (without chromosome 6), representing the scaled number of chromosomes with the predicted CNA present for each cell. Most of the blast cell clusters from AML patients have higher CNA count (colored in yellow and red) supporting malignant phenotype as compared to normal T cells with low CNA represented with cyan color.

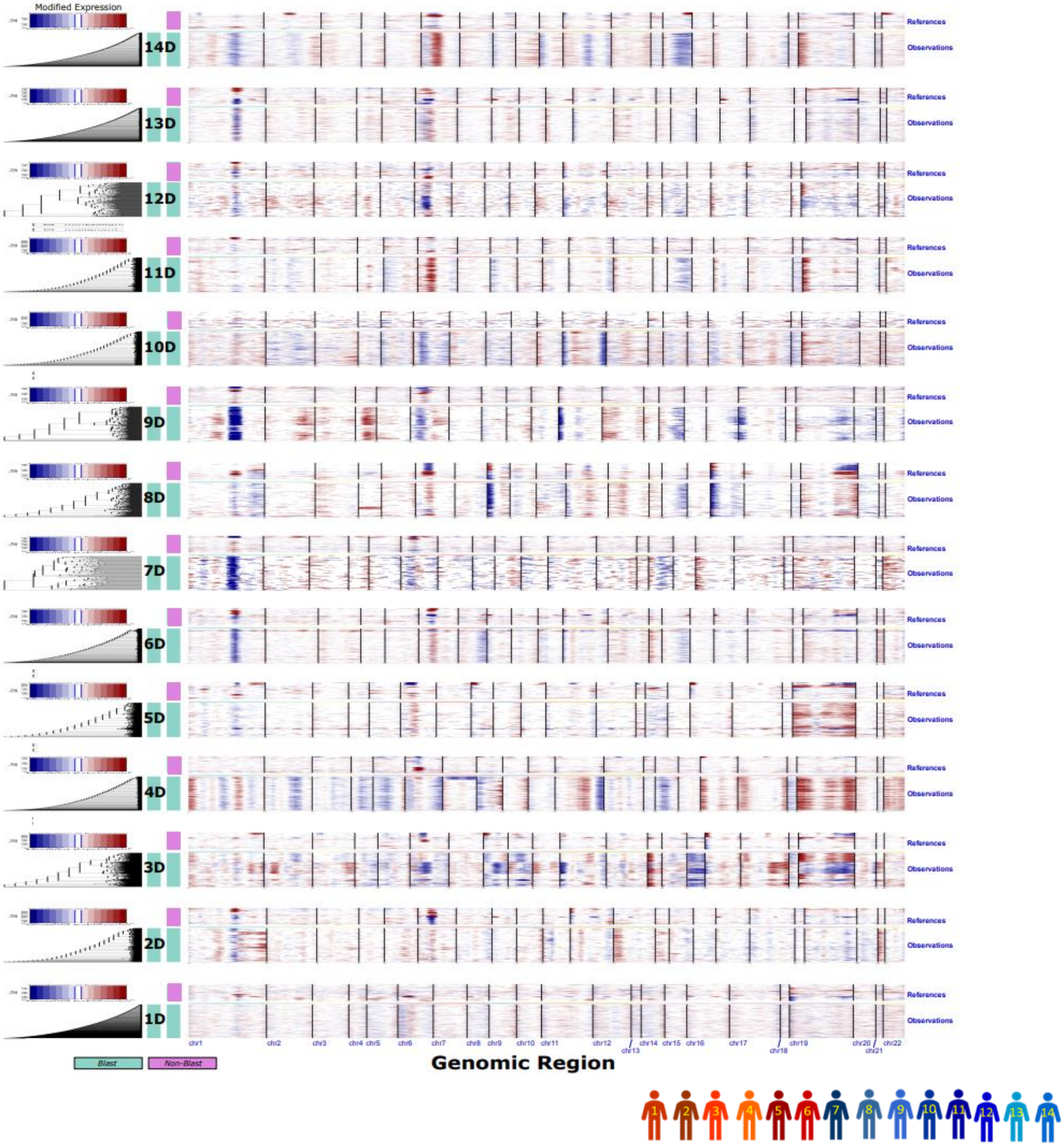

Supplementary Figure 17: CNA analysis of paired Dx AML-blast and non-blast cells from individual patients. Dx AML-blasts and non-blasts from patients 1-14 were analyzed using inferCNV tool[3]. The expression intensity of genes based on genomic locations in observational cells i.e., AML-blasts, was compared to reference cells i.e., matched AML non-blasts for same patient. Chromosomal amplifications (red) and deletions (blue) inferred across each chromosome are represented. The rows represent predicted CNAs in paired AML-blasts and reference cells from each patient. AML blast and non-blast cells are shown with cyan and pink color bars respectively. Columns represent genes ordered by chromosomal position. Dendrograms shown on the left show the hierarchical clustering of blast cells.

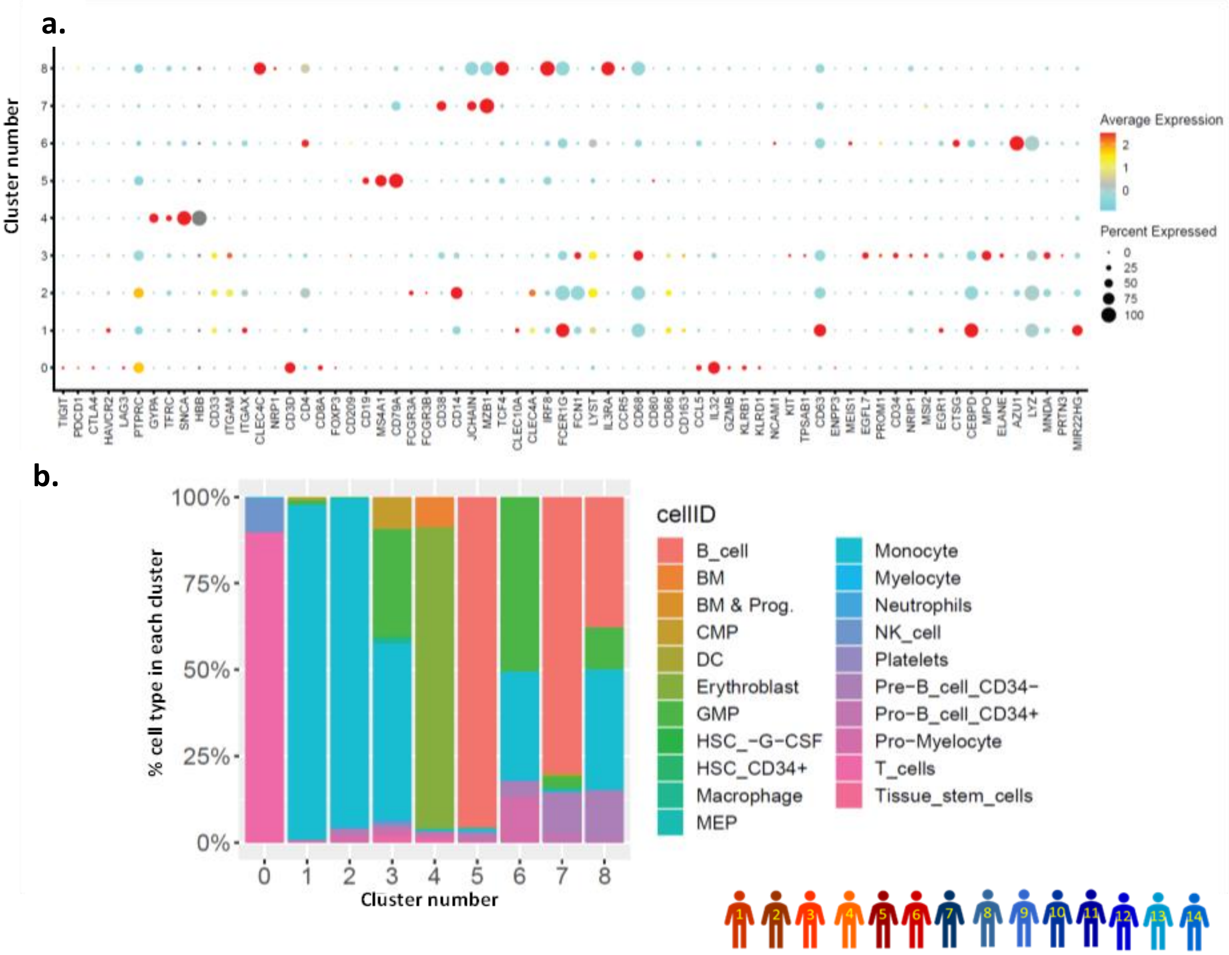

Supplementary Figure 18: Annotation of non-blasts BME clusters. Non-blast cells from Dx samples of patients 1-14 were selected and reclustered to perform focused analysis on non-blast cells. **a.** Expression of established gene markers used for annotating clusters. The X-axis shows gene names and Y-axis shows cluster numbers. Colour scale shows gene expression levels with red and cyan blue representing high and low expression respectively. The size of dot represents the percentage of cells expressing each gene in individual cell clusters. **b.** SingleR software-based automated annotation[1]. Combination of these two annotation methods was used to annotate the cell clusters: 0 - T cells (*CD3D, CD8A, IL32*), 1,2,6 - monocytes/macrophages (*CD4, CD14, CD63, FCER1G*), 3 - immature myeloid (*PROM1, MPO, ELANE*), 4 – erythrocyte (*GYPA, TFRC, SNCA*), 5 - B cells (*CD19, MS4A1, CD79A*), 7 – plasma (*JCHAIN, MZB1*), and 8 – pDC (*TCF4, IRF8*). Color key shows annotated cell type.

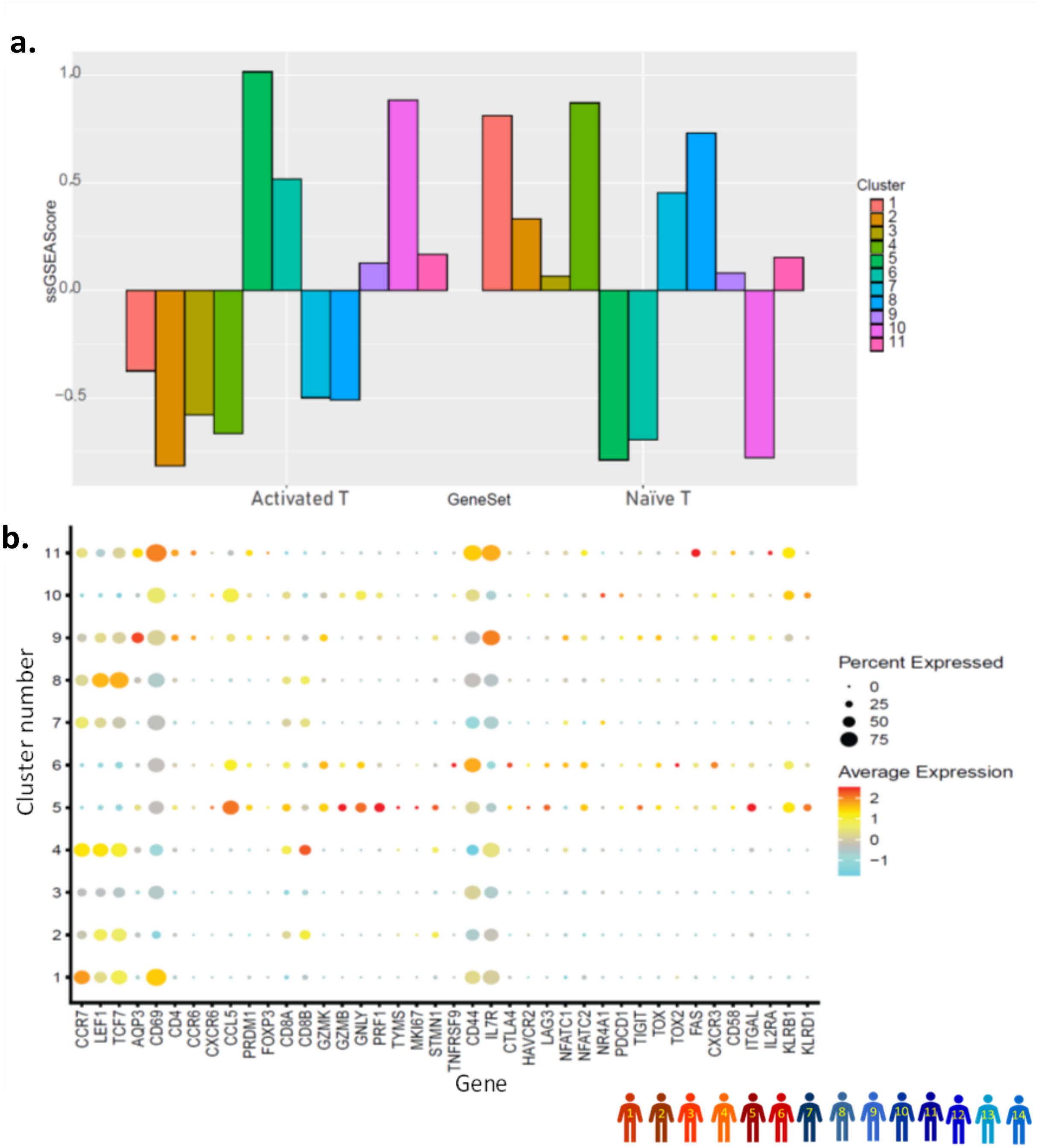

Supplementary Figure 19: Focused analysis on T cell clusters. T cells from Dx samples of patients 1-14 were subclustered for focused and detailed analysis. **a.** Scaled ssGSEA score based on activated T-cell (left) and naive T-cell (right) markers across T-cell subclusters. X-axis represents the subclusters. The genes used to perform ssGSEA analysis for naïve T cells were *CCR7, LEF1, TCF7* while the marker genes for T cell activation were *CCL5, KLRB1, KLRD1, GZMH, CD69, CD44.* **b.** Dot plot showing T cell sub-type marker gene expression. X-axis shows the genes and Y-axis the cell cluster number. Size of dots indicates percentage of cells in each cell cluster expressing the marker gene; color represents averaged scaled expression levels; cyan: low, yellow: medium and red: high.

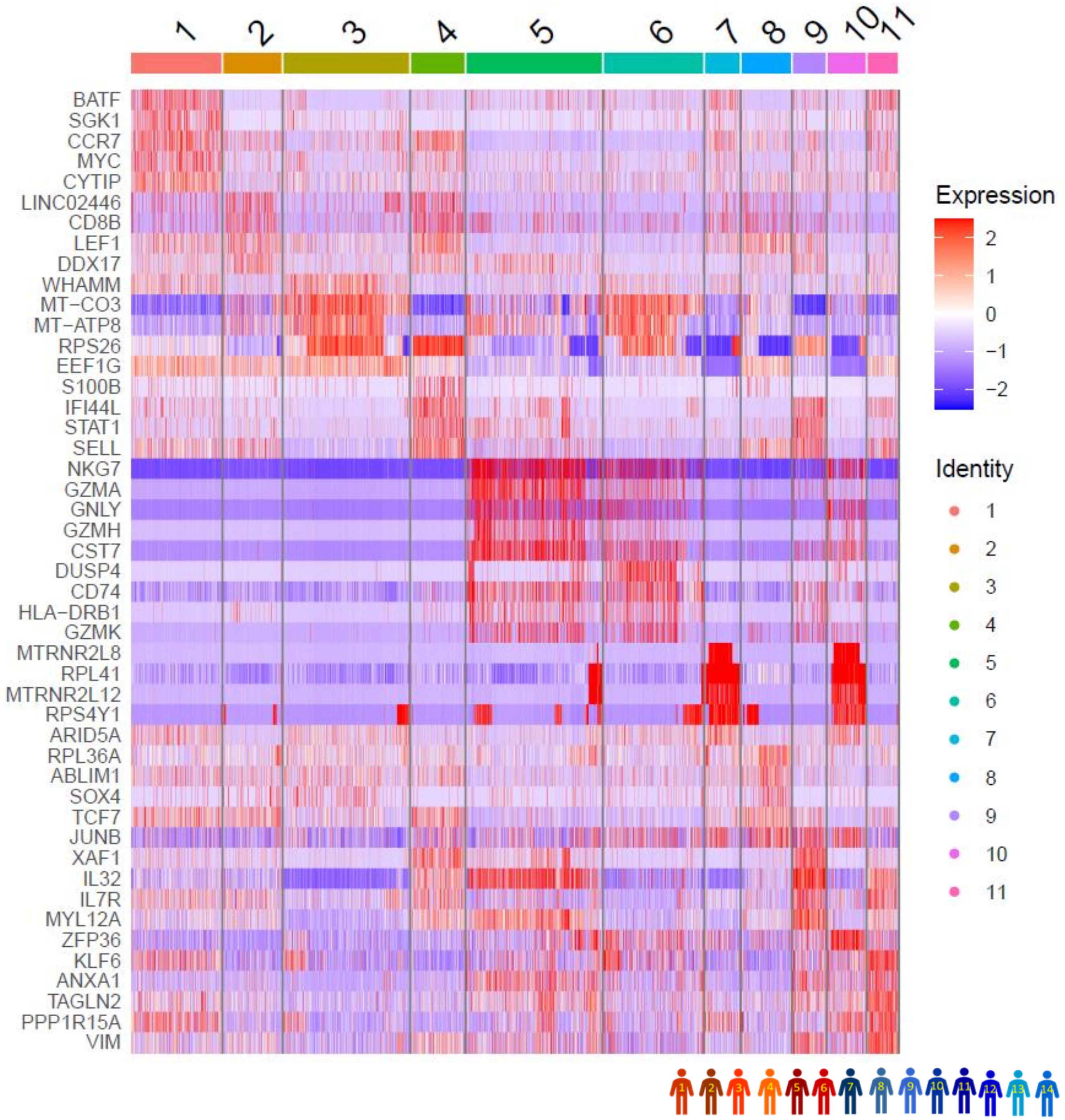

Supplementary Figure 20: Heatmap of differentially expressed genes for T cells subclusters. The T cells subclusters from Dx samples of patients 1-14 were analyzed for top DEGs between the clusters. The heatmap shows the top five, significantly (*P*<.05) overexpressed genes (by average log2FC) for each subcluster in the T-cell shown in **Fig. 4c**. Rows represent relative gene expression shown with blue and red colors representing low and high expression of genes, respectively. The columns represent the subcluster IDs for T cells.

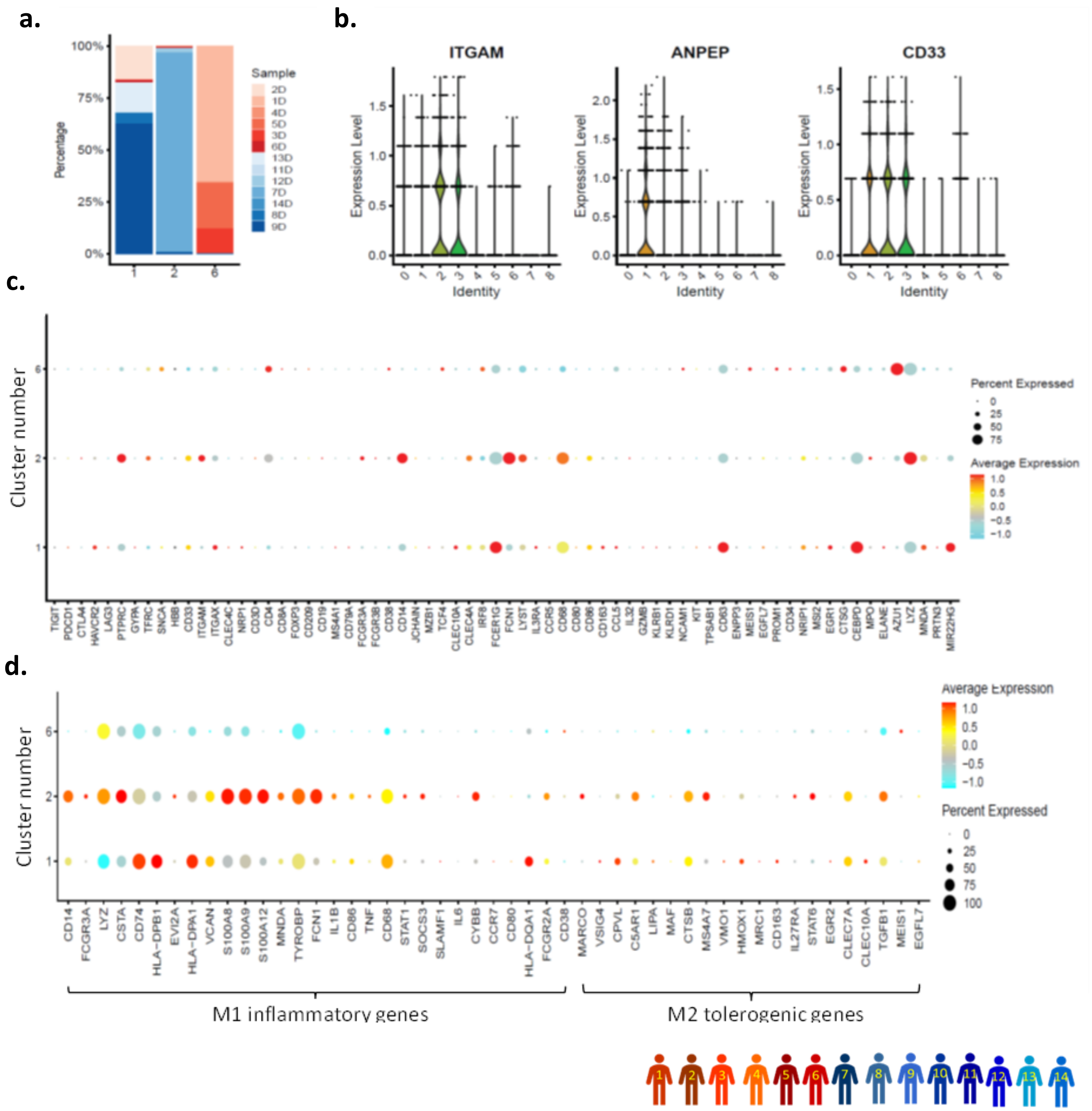

Figure S21: Detailed analysis on monocyte/macrophages from Dx samples. Monocytes/macrophages clusters (1, 2, 6) from relapse- and CCR-associated Dx samples of patients 1-14 were analyzed. **a.** Bar plot depicting patient representation in Monocytes/ Macrophages clusters. Cluster 1 and 2 are enriched with cells from patients with CCR whereas cluster 6 is enriched with cells from relapse patients. X-axis shows cluster number and Y-axis the percentage of cells from each sample in a cluster. **b.** Expression of leukemia myeloid marker genes in the three Monocyte/macrophages clusters**.** X-axis represents cluster number (identity) and Y-axis the gene expression levels. **c.** Dot plot showing expression of canonical markers genes used for annotating the clusters. **d.** Expression of M1 and M2 marker gene markers in clusters 1, 2 and 6. X-axis shows genes, and Y-axis shows the cluster numbers. Colour scale shows gene expression levels with red and cyan blue representing high and low expression respectively. The size of dot represents the percentage of cells expressing each gene in individual cell clusters.

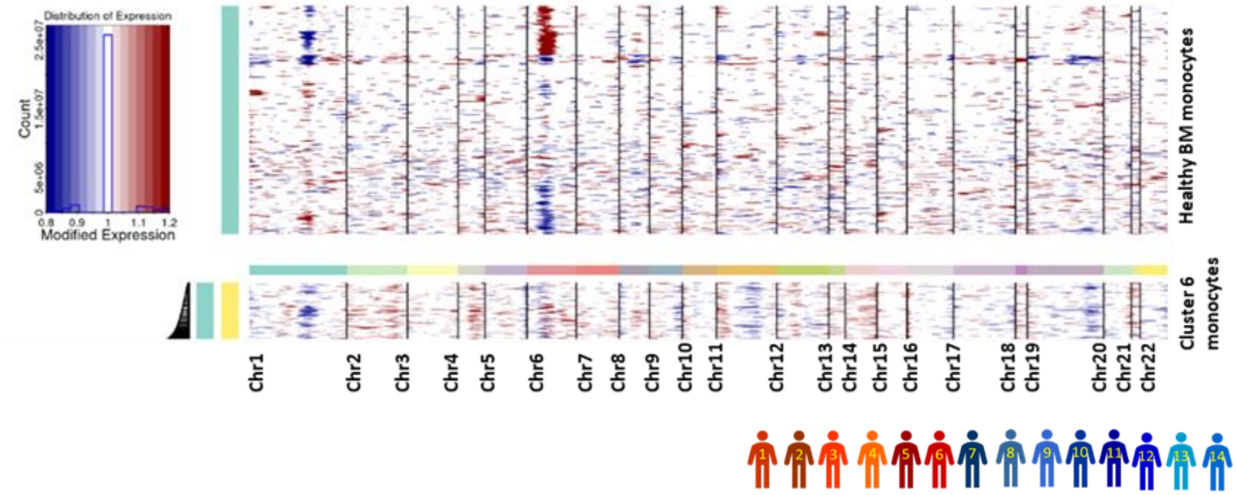

Supplementary Figure 22: CNA analysis of monocyte/macrophage cluster 6 predominantly present in relapse patient samples. Monocytes/ macrophages cluster 6 present in Dx AML samples (from patients 1-14), characterized by high expression of premature genes like *AZU1, LYZ,* and *KCNE5* was tested for CNAs. The inferCNV tool[3] was used to analyze the expression intensity of genes based on genomic locations in observational cells (cluster 6) in comparison to reference cells (healthy BM monocytes). Chromosomal amplifications and deletions are shown in red and blue colors respectively. Columns correspond to genes ordered by chromosomal positions.

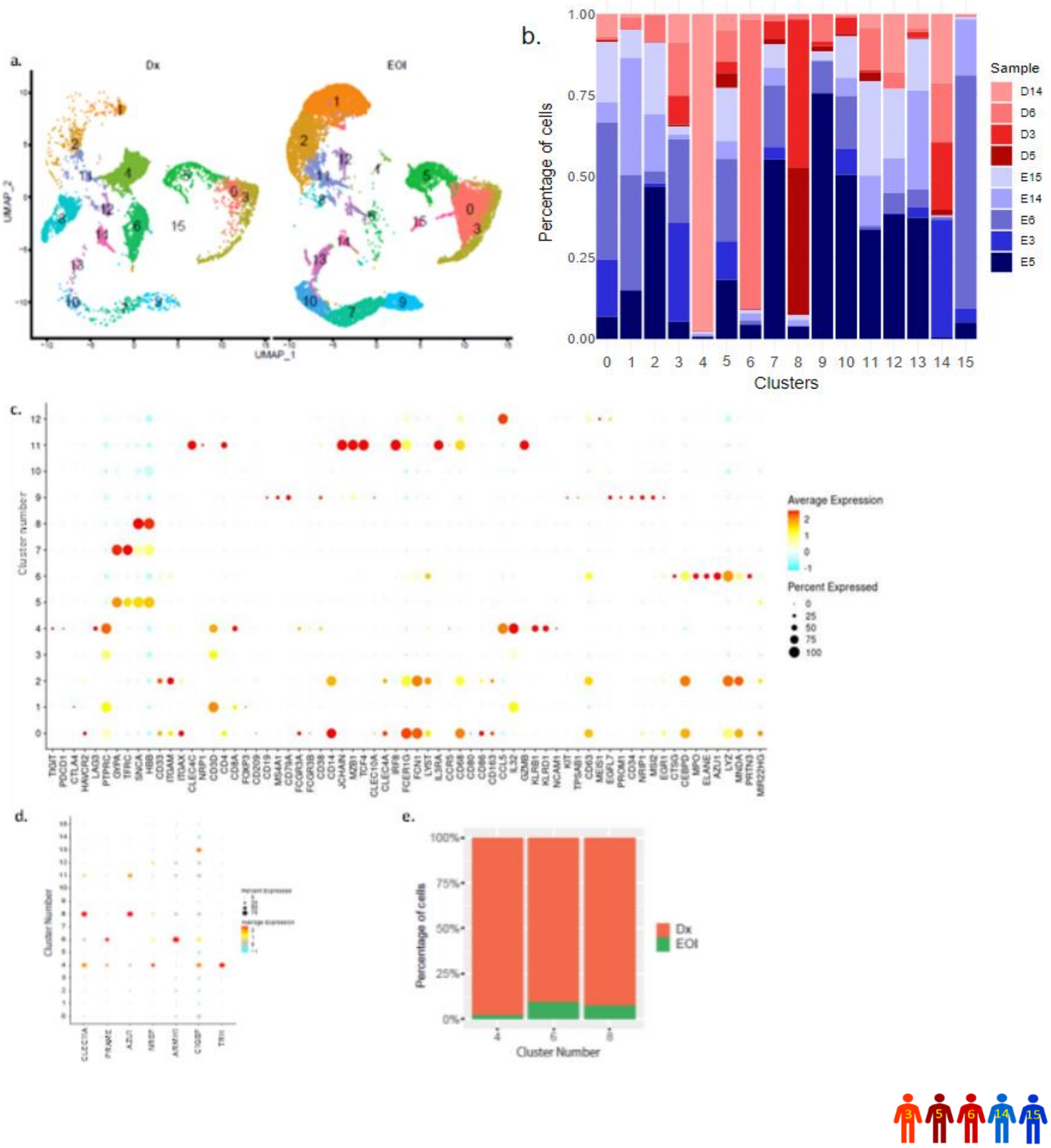

Supplementary Figure 23: Identification of residual blast clusters using Dx, and EOI samples. Integrated analysis of matched Dx, EOI samples (patients 3,5,6,14) as well as one unmatched EOI samples (patient 15) to identify residual blast cells in EOI samples. **a.** Split UMAP plots show the putative blast cells (clusters 4, 6, 8) that are significantly over-represented in Dx samples and reduced in the EOI samples. **b.** Bar plot showing percentage of individual Dx and EOI samples in each cluster. Blue colors represent EOI samples and red colors represent Dx samples. **c.** Dot plot showing expression of canonical markers genes used for annotating the clusters. **d.** Dot plot of 7-gene AML-blast signature shows over-expression in clusters 4,6, and 8. X-axis represents gene name and Y- axis represents cluster number. **e.** Bar plot of blast clusters showing percentage of Dx and EOI cells per cluster.

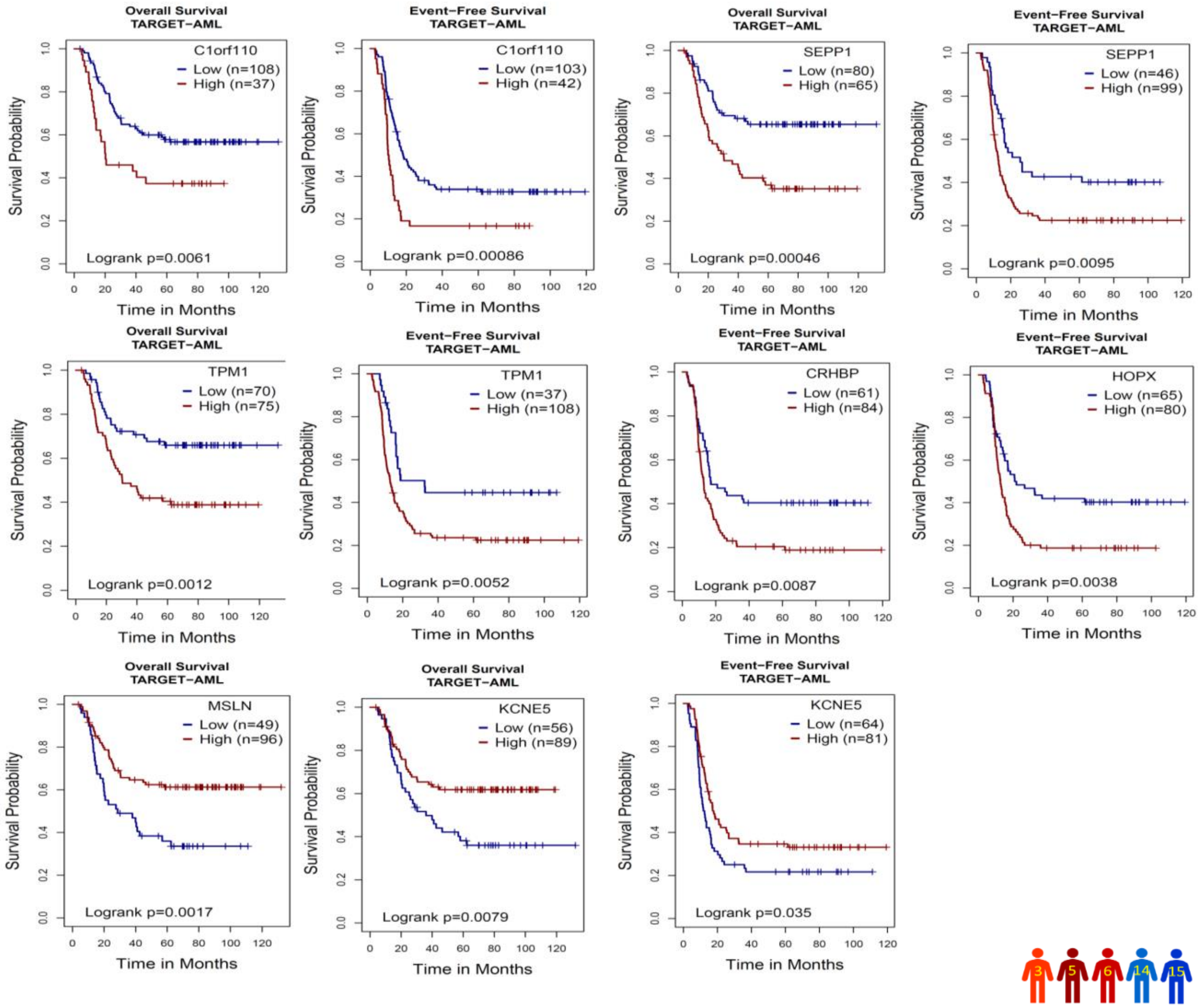

Supplementary Figure 24: Survival analysis of differentially expressed genes in the residual blast cell clusters. Kaplan Meir survival curves show that elevated expression of residual blasts enriched genes such *as FAM30A/C1orf110, SELENOP/SEPP1, TPM1* are associated with poorer OS and EFS while *CRHBP* and *HOPX* were associated with lesser EFS. Similar analysis on genes downregulated in the residual blast cell depicted significantly better OS (*MSLN* and *KCNE5*) and EFS (*KCNE5*).

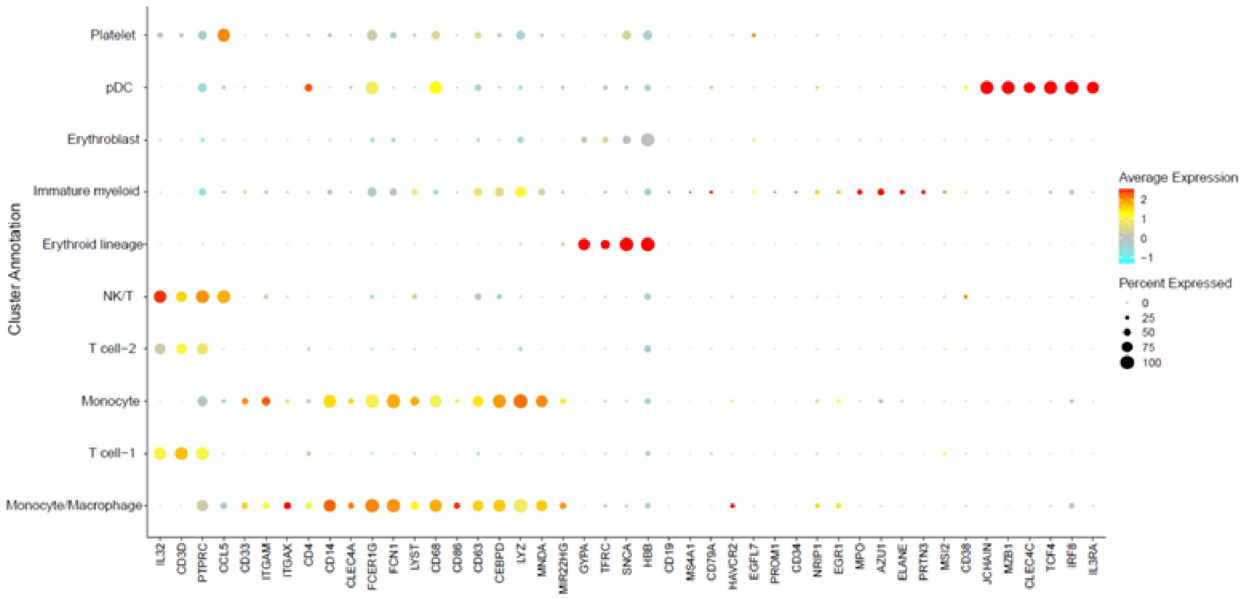

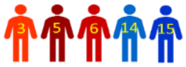

Supplementary figure 25: Annotation of cell clusters from EOI samples. Cells (n=15,070) from only post-therapy EOI samples (patients 3, 5, 6, 14, 15) were isolated from integrated Dx, EOI data object and re-clustered for focused analysis. Dot plot of canonical markers used for cluster annotation with genes on X-axis and cell types on Y-axis. Colour scale shows gene expression levels with red and cyan blue representing high and low expression respectively. The size of dot represents the percentage of cells expressing each gene in individual cell types.

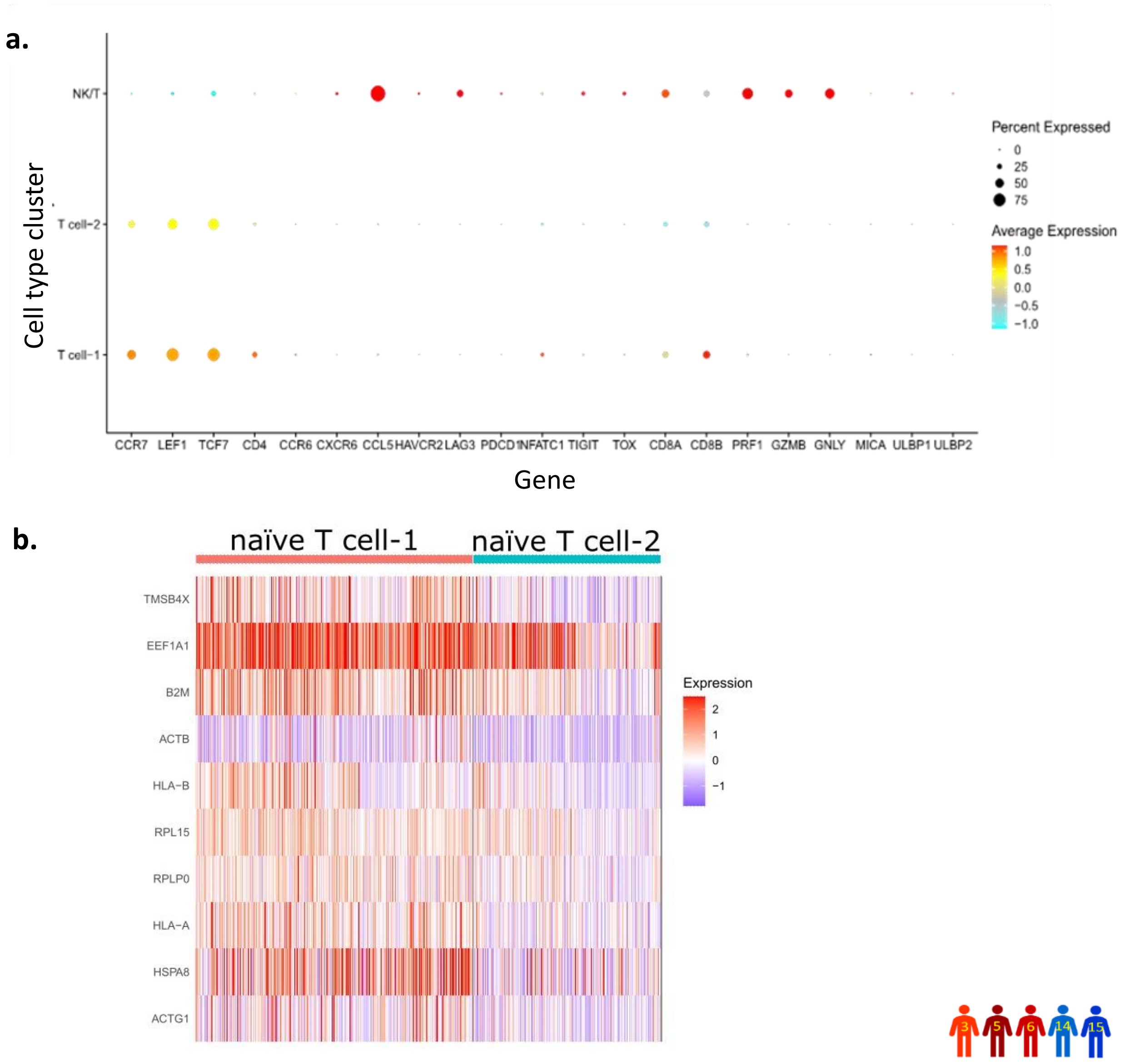

Supplementary Figure 26: Detailed analysis on T cell analysis clusters of EOI samples. In this analysis EOI T cells from the patients with relapse (3E, 5E, 6E) and with CCR (14E, 15E) were analyzed. **a.** Dot plot of the three T cells and NKT cell clusters. The X-axis shows the genes, Y-axis is the cell type cluster. Size of dots indicates the percentage of cells in each cell cluster expressing the marker gene; color represents averaged scaled expression levels; cyan: low, yellow: medium, red: high. T cell-1 cluster (higher in patients with CCR) expresses naïve T cell markers at a higher level than T cell-2 cluster (higher in patients with relapse) while NK/T cluster expresses exhausted and cytotoxic markers. **b.** Heatmap shows top differentially expressed genes revealed upon comparing T cell-2 cluster to T cell-1 cluster. Color scale represents gene expression with red and blue being high and low expression respectively.

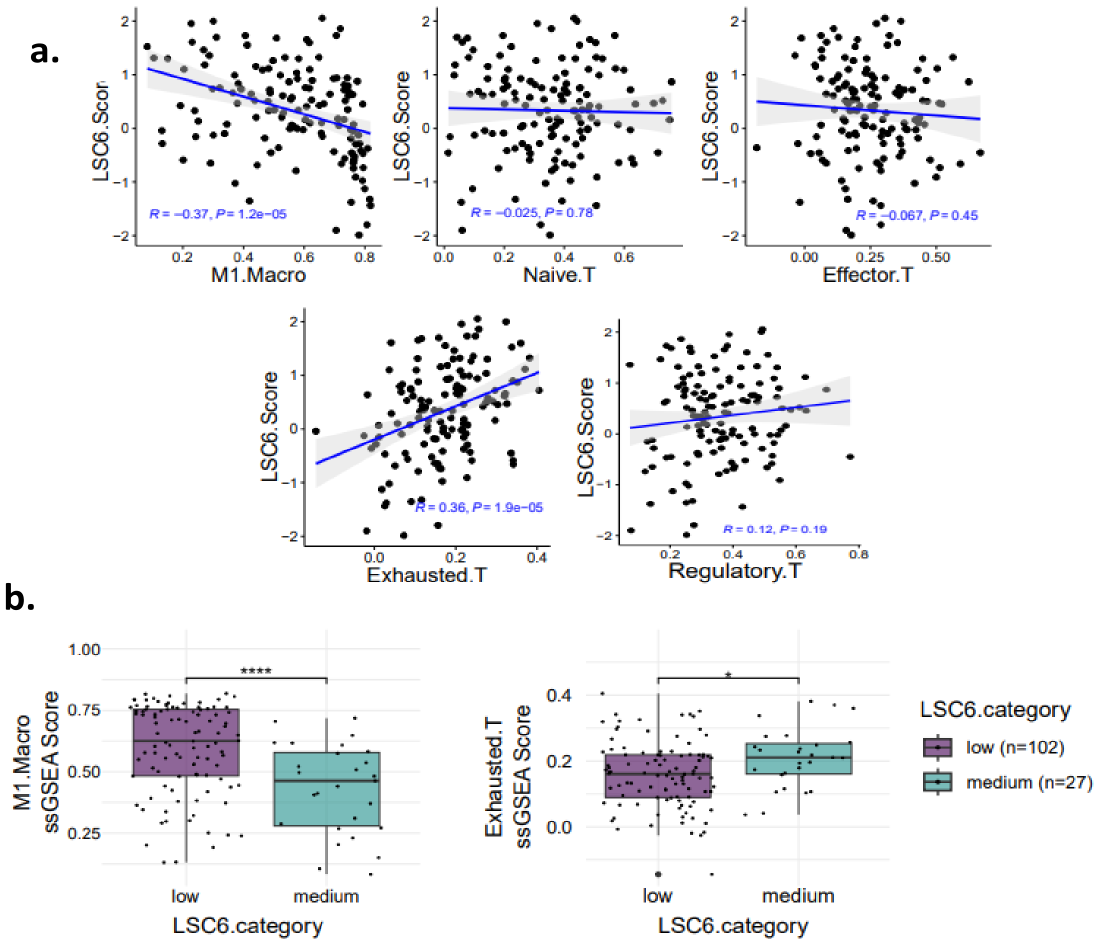

Supplementary Figure 27: Gene set enrichment analysis of bulk RNA-seq data to explore the association of different immune cell types and AML risk scores. The bulk RNA-seq dataset from Fornerod et al., AML dataset[6] (n=132) was used to validate the associations of immune cells with clinical outcomes based on single-cell analysis. Fornerod et al., AML dataset contained LSC6 scores and risk categories for each of the samples. The immune cell signatures from single cell data were used to calculate enrichment using the ssGSEA algorithm. The immune cell signatures are: M1 Macrophage (*S100A8, S100A9, S100A12, TYROBP, VCAN, CD68, MNDA, CYBB, STAT1*), naïve T-cell (*CCR7, LEF1, TCF7, SELL*), effector T-cell (*CCL5, NKG7, GNLY, GZMA, GZMK, NFACT1*), exhausted T-cell (*HAVCR2, LAG3, PDCD1, NFATC1, TIGIT, TOX*), and regulatory T-cell (*CCL5, KLRB1, KLRD1, GZMH, CD69, CD44*). **a.** Scatter plots showing the correlation of LSC6 scores and ssGSEA scores for different immune cell signatures. Pearson's correlation coefficient and corresponding *P*-values are calculated to determine the significance of associations. **b.** Boxplots showing the ssGSEA scores for M1 Macrophages and exhausted-T cells for AML risk category groups calculated based on LSC6 score. Wilcoxon rank sum test (two-tailed) was used to perform comparisons among different AML risk groups i.e., low- (n=102) and medium-risk (n=27) biologically independent sample groups (high-risk group was excluded from analysis due to small number of samples (n=3)). The significance of association was represented as *P*-values (**** *P*<.0001, * *P*<.05). Boxplots show the distribution of scores with the center of the box representing the median, upper and lower bounds representing 75% and 25% percentiles, and upper and lower whiskers extending to the largest value no further than 1.5 * interquartile range) from bounds of box. Source data are provided as a Source Data file.

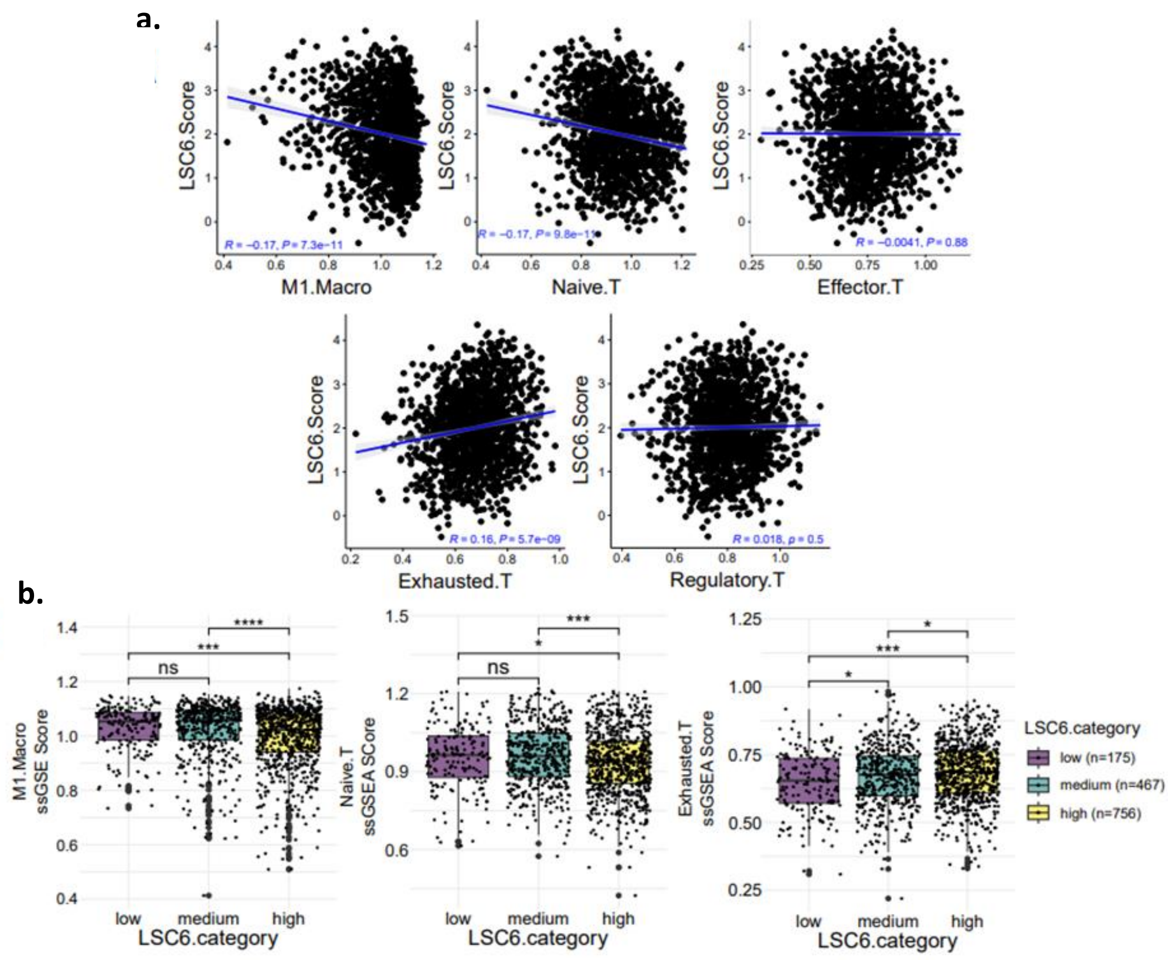

Supplementary Figure 28: Gene set enrichment analysis of TARGET AML-2 data to explore the association of different immune cell types with AML risk score. The bulk RNA-seq dataset of TARGET AML-2 (n=1,398) was used to assess the validity of our immune signature findings in single-cell assays. LSC6 scores were calculated, and risk categories were assigned for each sample. The immune cell signatures from single-cell data were used to calculate enrichment using ssGSEA algorithm. The immune cell signatures are: M1 Macrophage (*S100A8, S100A9, S100A12, TYROBP, VCAN, CD68, MNDA, CYBB, STAT1*), naïve T-cell (*CCR7, LEF1, TCF7, SELL*), effector T-cell (*CCL5, NKG7, GNLY, GZMA, GZMK, NFACT1*), exhausted T-cell (*HAVCR2, LAG3, PDCD1, NFATC1, TIGIT, TOX*), and regulatory T-cells (*CCL5, KLRB1, KLRD1, GZMH, CD69, CD44*). **a.** Scatter plots showing the correlation of LSC6 scores and ssGSEA scores for different immune cells. Pearson's correlation coefficient and corresponding *P*-values were calculated to determine the significance of associations. **b.** Box plots showing the ssGSEA scores for M1 Macrophages, naïve T-cells, and exhausted T-cells with AML risk groups calculated based on LSC6 score. The Wilcoxon rank sum test (two-tailed) was used for comparisons among different AML risk groups (i.e., low (n=175), medium (n=467), high (n=756) biologically independent samples). The significance of association was represented as *P*-values (**** *P*<.0001; *** *P*<.001; * *P*<.05; ns= *P*>.05). Boxplots show the distribution of scores with the center of the box representing the median, upper, and lower bounds representing 75% and 25% percentiles, and upper and lower whiskers extending to the largest value no further than 1.5 * interquartile range) from bounds of box. Source data are provided as a Source Data file.

Supplementary Tables

Supplementary Table 1: Patients clinical and genetic information.

Patient No.	Sample type used			age (years)	PB Blast (%)	BMA Blast (%)	AML WHO Classification	Mutations, rearrangements							
	Dx	EOI	Rel					-7/add(7q)/del(7q)	t(8;21) RUNX1	t(16;16)(p13.1q22)	MLL rearrangement (KMT2A)	CCAAT / CEBPA	FLT3 /ITD	Allelic ratio	Nucleophosmin (NPM)
1	1D			12	90.5	96.4	AML with myelodysplasia-related changes	Negative	Negative	Negative	Positive	Negative	No		Negative
2	2D			13	25.0	68.6	AML with myelodysplasia-related changes	Negative	Negative	Negative	Negative	Negative	No		Negative
3	3D	3E		6.3	21.0	85.8	AML, NOS	Negative	Negative	Negative	Negative	Negative	No		Negative
4	4D			1.5	0.0	59.0	AML, NOS	Negative	Negative	Negative	Positive	Negative	No		Negative
5	5D	5E	5R	17.8	83.0	91.1	AML, NOS	Negative	Negative	Negative	Positive	Negative	No		Negative
6	6D	6E	6R	10.8	8.0	91.2	AML with myelodysplasia-related changes	Positive	Negative	Negative	Negative	Negative	No		Negative
7	7D			1.6	85.6	81.0	AML with minimal differentiation	Negative	Negative	Negative	Negative	Negative	No		Negative
8	8D			12.3	84.1	89.8	AML with mutated CEBPA	Negative	Negative	Negative	Negative	Positive	No		Negative
9	9D			1.6	37.0	66.2	AML with inv(16)(p13.1q22) or t(16;16)(p13.1;q22), CBFB/MYH11	Negative	Negative	Positive	Negative	Negative	No		Negative
10	10D			12.1	95.7	96.4	Acute myeloid leukemia with mutated NPM1	Negative	Negative	Negative	Negative	Negative	No		Positive
11	11D			7.7	27.0	29.0	Acute myeloid leukemia, t(8;21)(q22;q22) RUNX1-RUNX1T1	Negative	Positive	Negative	Negative	Negative	No		Negative
12	12D			17.5	32.5	29.5	AML, t(8;21)(q22;q22) RUNX1-RUNX1T1	Negative	Positive	Negative	Negative	Unknown or N/A	No		Negative
13	13D			17.3	53.0	65.8	AML with inv(16)(p13.1q22) or t(16;16)(p13.1;q22), CBFB/MYH11	Negative	Negative	Positive	Negative	Positive	No		Positive
14	14D	14E		16.7	10.0	40.3	AML, t(8;21)(q22;q22) RUNX1-RUNX1T1	Negative	Positive	Negative	Negative	Negative	No		Negative
15		15E		16.4	71.0	Not done	AML, NOS	Negative	Negative	Negative	Negative	Negative	Yes	0.5	Positive
16	16D	16E		17.6	18.0	40.3	AML with myelodysplasia-related changes	Positive	Negative	Negative	Negative	Negative	No		Negative
17	17D	17E		1.5	3.0	92.8	Acute megakaryoblastic leukemia	Negative	Negative	Negative	Negative	Negative	No		Negative
18	18D	18E		16.1	5.0	88.8	APL (AML with t(15;17)(q22;q12)) PML/RARA	Unknown or N/A	Unknown or N/A	Unknown or N/A	Unknown or N/A	Negative	No		Negative
19	19D	19E		14.8	45.7	90.8	AML with mutated RUNX1	Negative	Negative	Negative	Negative	Negative	Yes	0.42	Negative
20	20D	20E		6.8	55.0	75.4	AML, t(8;21)(q22;q22) RUNX1-RUNX1T1	Positive	Positive	Negative	Negative	Negative	N/A		Negative

PB: Peripheral blood, BMA: Bone marrow aspirate. Samples from patients 3,5,6,14 were used for developing the 7-gene signature. Samples from patients 1-14 were used for diagnosis (Dx) blast and non-blast analysis. Matched Dx, end of induction (EOI) samples from patient 3,5,6,14 and EOI sample from patient 15 were used for EOI blast and non-blast analysis. Paired Dx, EOI samples from patients 16-20 were used for 7-gene signature validation. AML genetic subtype DEGs analysis was conducted on all the Dx samples i.e., from patients 1-14, 16-20 and CNA analysis was carried out on Dx blasts of patients 1-14.

Supplementary Table 2: Clinical risk category, MRD status, and treatment regimen of AML patients with relapse and CCR.

Patient No.	EOI Stratification	EOI MRD	Patient relapsed?	Alive/ Deceased	Induction Regimen
1	High risk	Positive	Yes	Deceased	AAML 1031 (on study)
2	Low risk	Negative	Yes	Deceased	Institutional modification, AAML1031
3	Low risk	Negative	Yes	Deceased	Institutional AML protocol
4	Low risk	Negative	Yes	Alive	AAML 1031 (on study)
5	Low risk	Negative	Yes	Deceased	AAML1031 NOS
6	Low risk	Negative	Yes	Alive	Institutional AML protocol
7	Low risk	Negative	No	Alive	AAML 1031 NOS
8	Low risk	Negative	No	Alive	Institutional AML protocol
9	Low risk	Negative	No	Alive	AAML1031 NOS
10	Low risk	Negative	No	Alive	per AAML 1031 Arm A
11	Low risk	Negative	No	Alive	AAML 1031 NOS
12	Low risk	Negative	No	Alive	AAML1031 NOS
13	N/A	N/A	No	Deceased	AAML 1031 (ADE)
14	Low risk	Negative	No	Alive	AAML1031 NOS
15	High risk	Negative	No	Alive	AAML 1031 (on study)
16	High risk	Negative	No	Alive	AAML1031
17	High risk	positive	No	Deceased	AAML0531
18	Low risk	Negative	No	Alive	AALL1331
19	High risk	Negative	No	Alive	AAML0531, AAML1031
20	Low risk	Negative	No	Alive	AAML0531

MRD: Measurable/ minimal residual disease, Dx: diagnosis, EOI: end of induction, CCR: Continuous clinical remission

Supplementary Table 3: Forty-four genes showing significant fold change in high blast % versus low blast % samples and high blast % versus EOI samples in the TARGET AML data set 1 (TARGET AML-1).

Gene	High vs. low blast %		High blast % vs. EOI	
	Fold change	P value	Fold change	P value
MSLN	347.2	0	194.3	2.60E-09
RPS17	345.9	0	325.2	4.11E-53
PRAME	178.3	0	48.0	6.78E-07
C1QTNF4	74.1	0	19.8	2.36E-09
TRH	39.9	0	8.5	0.002285
CITED4	8.9	0	15.2	1.67E-19
CLEC11A	8.5	0	6.5	1.79E-10
MARCKSL1	8.1	0	4.1	1.31E-09
KCNE5	7.2	0.04	13.5	0.000447
HMGA1	6.5	0	6.0	1.43E-22
AZU1	6.4	0	3.8	0.000493
CFD	6.4	0	3.0	0.000917
NPW	6.3	0	5.1	5.04E-06
FABP5	4.9	0	4.2	3.85E-15
RUNX1	4.8	0	3.7	6.44E-15
SRM	4.8	0	4.8	7.00E-16
EBPL	4.4	0	3.5	8.48E-13
C1QBP	4.3	0	4.8	6.05E-27
PPP1R27	4.2	0.03	3.4	0.025676
PTBP1	3.6	0	3.2	3.17E-20
PDLIM1	3.4	0	2.5	0.002042
HLA-DPA1	3.3	0	6.2	2.28E-09
EEF2	3.3	0	2.4	4.50E-11
MYB	3.1	0	2.5	2.06E-05
CALR	3.1	0	2.1	2.42E-06
HLA-DQA1	3.0	0.01	8.0	3.80E-07
MFSD10	2.8	0	2.8	1.40E-12
ARMH1	2.7	0	1.8	0.002773
RPS24	2.6	0	2.5	2.76E-13
HMGN1	2.6	0	2.4	9.74E-14
CAPRIN1	2.5	0	2.2	1.34E-12
RPL7A	2.4	0	2.2	3.79E-13
NME4	2.3	0	2.2	5.16E-08
GRK2	2.3	0	2.3	6.58E-11
TUBB	2.3	0	2.1	3.49E-07
TYMS	2.3	0	2.3	9.87E-06
RPL4	2.3	0	2.0	5.44E-10
KHDRBS1	2.2	0	2.1	1.88E-12
SLC25A1	2.2	0	1.9	0.000533
P4HB	2.2	0	1.8	4.76E-05
SNHG7	2.1	0	2.1	6.08E-05
PHB2	2.1	0	2.0	3.48E-11
NREP	1.9	0.03	1.9	0.005117
GSN	1.8	0.03	3.2	7.43E-07

Differentially expressed genes identified using the limma method (linear model using weighted least squares, two-tailed, *P*-values adjusted with Benjamini-Hochberg). Biologically independent samples in high blasts % group, n=203; low blasts % group, n=14; EOI group, n=24.

Supplementary Table 4: Fold change of forty-four genes in Dx AML-blast clusters as compared to EOI non-blast clusters.

Gene	Fold change	Adjusted P value
AZU1	3.0	0
CLEC11A	2.2	0
TRH	1.9	0
ARMH1	1.8	0
TUBB	1.8	0
C1QTNF4	1.8	0
CFD	1.8	1.80E-114
NPW	1.7	0
HLA-DPA1	1.7	0
CALR	1.7	0
PPP1R27	1.6	0
HMGN1	1.6	0
HMGA1	1.6	0
SNHG7	1.5	0
PDLIM1	1.5	0
KCNE5	1.4	0
RPL7A	1.4	0
C1QBP	1.4	0
GSN	1.4	1.03E-296
P4HB	1.4	3.13E-254
NREP	1.4	0
RPS24	1.4	0
FABP5	1.3	0
TYMS	1.3	0
PRAME	1.3	0
RPS17	1.3	2.09E-281
EEF2	1.3	8.16E-260
MSLN	1.3	0
CITED4	1.3	1.32E-216
CAPRIN1	1.3	7.11E-298
MARCKSL1	1.3	1.90E-217
PHB2	1.3	1.57E-238
EBPL	1.3	0
HLA-DQA1	1.3	0
KHDRBS1	1.3	2.91E-210
PTBP1	1.3	2.58E-153
NME4	1.3	3.13E-271
MYB	1.3	0
RUNX1	1.2	3.28E-241
SLC25A1	1.2	1.22E-284
MFSD10	1.2	2.76E-200
GRK2	1.2	1.94E-98
RPL4	1.2	3.40E-186
SRM	1.2	2.79E-179

Differentially expressed genes identified using the *FindMarkers* function of the Seurat package (two-sided Wilcoxon rank sum test, Bonferroni correction adjusted *P*-value).

Supplementary Table 5: List of twenty genes overexpressed in Dx AML-blast clusters.

Gene	Fold change	Adjusted P value
AZU1	3.0	0
CLEC11A	2.2	0
TRH	1.9	0
ARMH1	1.8	0
C1QTNF4	1.8	0
NPW	1.7	0
PPP1R27	1.6	0
KCNE5	1.4	0
C1QBP	1.4	0
NREP	1.4	0
FABP5	1.3	0
PRAME	1.3	0
MSLN	1.3	0
CITED4	1.3	1.32E-216
CAPRIN1	1.3	7.11E-298
EBPL	1.3	0
MYB	1.3	0
RUNX1	1.2	3.28E-241
SLC25A1	1.2	1.22E-284
MFSD10	1.2	2.76E-200

Differentially expressed genes identified using the *FindMarkers* function of the Seurat package (two-sided Wilcoxon rank sum test, Bonferroni correction adjusted *P*-value).

Supplementary Table 6: Survival analysis of the twenty blasts-overexpressed genes in the TARGET AML dataset 1 (TARGET AML-1).

Gene	Grouping method: cutpoint (cut-point | P-value | percentile)	Defined groups #Low|#High	Log Rank p-value | HR P-value	Hazard Ratio, HR (95% CI)	Survival analysis
AZU1	cutp: 7.14|0.722|0.352	51|94	0.167|0.169	1.44 (0.856-2.43)	Overall
CLEC11A	cutp: 8.93|0.54|0.841	122|23	0.0197|0.022*	1.96 (1.1-3.48)	Overall
TRH	cutp: 3.45|0.00239*|0.566	82|63	0.000396|0.000626***	0.396 (0.233-0.674)	Overall
C1orf228 (ARMH1)	cutp: 5.12|0.0279*|0.607	88|57	0.00296|0.00359**	2.03 (1.26-3.27)	Overall
C1QTNF4	cutp: 5.75|0.354|0.648	94|51	0.0944|0.0971	0.643 (0.381-1.08)	Overall
NPW	cutp: 3.9|0.0746|0.572	83|62	0.0132|0.0147*	0.531 (0.319-0.883)	Overall
PPP1R27	cutp: 0.46|0.49|0.152	22|123	0.0141|0.0162*	0.493 (0.277-0.878)	Overall
KCNE5	cutp: 0.348|0.0389*|0.386	56|89	0.00792|0.009**	0.53 (0.329-0.853)	Overall
C1QBP	cutp: 6.59|0.778|0.91	132|13	0.0416|0.046*	2.05 (1.01-4.14)	Overall
NREP	cutp: 3.78|0.0404*|0.586	85|60	0.00546|0.00637**	1.95 (1.21-3.14)	Overall
FABP5	cutp: 3.28|0.0854|0.366	53|92	0.0137|0.0155*	1.95 (1.14-3.34)	Overall
PRAME	cutp: 0.0107|0.817|0.0429	11|134	0.0778|0.0963	3.3 (0.808-13.5)	Overall
MSLN	cutp: 0.333|0.0155*|0.329	49|96	0.00168|0.00214**	0.473 (0.293-0.763)	Overall
CITED4	cutp: 4.3|0.764|0.179	26|119	0.0765|0.0796	0.605 (0.345-1.06)	Overall
CAPRIN1	cutp: 4.76|0.000129*|0.441	64|81	3.18e-05|7.31e-05***	2.99 (1.74-5.14)	Overall
EBPL	cutp: 5.26|0.921|0.614	89|56	0.395|0.395	1.23 (0.762-1.99)	Overall
MYB	cutp: 4.63|6.58e-05*|0.448	65|80	1.05e-05|3.04e-05***	3.23 (1.86-5.61)	Overall
RUNX1	cutp: 3.92|0.133|0.186	27|118	0.007|0.0103**	3 (1.3-6.94)	Overall
SLC25A1	cutp: 4.07|0.87|0.455	66|79	0.25|0.251	1.33 (0.818-2.15)	Overall
MFSD10	cutp: 5.47|0.00818*|0.579	84|61	0.00147|0.00189**	2.13 (1.32-3.44)	Overall

Gene symbols are in bold . * Symbol indicates one-sided log-rank and Wald test significance at * *P*<.05, ** *P*<.01, *** *P*<.001. The number of patients is specified under "defined groups #Low I #High".

Supplementary Table 7: AML-blasts 7-gene signature.

Gene	Ensemble ID	Fold change in gene expression			Survival analysis Hazard Ratio (95% CI)	Function associated with gene
		Dx AML-blasts vs. EOI blast cells*	Dx High blast% vs Low blast% samples**	Dx high blast% vs EOI samples**		
CLEC11A	ENSG00000105472	2.15	2.17	2.55	1.96<br>(1.1 – 3.48)	Growth factor for primitive hematopoietic progenitor cells
PRAME	ENSG00000185686	1.34	0.93	6.38	3.3<br>(0.81 - 13.5)	Promote cancer cell growth via repressor of retinoic acid receptor
AZU1	ENSG00000172232	3	0.5	2.22	1.44<br>(0.86 - 2.43)	Multifunctional inflammation Mediator
NREP	ENSG00000134986	1.36	1.14	1.07	1.95<br>(1.21 - 3.14)	Plays a role in neural function, augments motility of gliomas.
ARMH1	ENSG00000198520	1.84	1.29	1.24	2.03<br>(1.26 - 3.27)	Not identified yet
C1QBP	ENSG00000108561	1.4	1.04	1.94	2.05<br>(1.01-4.14)	Regulation of apoptosis and splicing
TRH	ENSG00000170893	1.93	0.97	2.17	0.396<br>(0.23 - 0.67)	Controls the secretion of thyroid-stimulating hormone

Gene expression fold change based on *scRNA-seq data from four Dx, EOI samples (patients 3,5,6,14) and **TARGET AML dataset 1 (TARGET AML-1).

Supplementary Table 8: Survival analysis of gene signatures.

Gene set	Grouping method: cutpoint (cut-point | p value | percentile)	Defined groups #Low|#High	Log Rank p-value | HR p-value	Hazard Ratio, HR (95% CI)	Survival analysis
2-genes signature	cutp: 1.12|0.00673*|0.738	107|38	0.00015|0.00025***	2.5 (1.5-4.1)	Overall
2-genes signature	cutp: 0.193|0.00524*|0.469	68|77	0.0012|0.0015**	1.9 (1.3-2.9)	Event-Free
7-genes signature	cutp: 1.58|0.506|0.876	127|18	0.0073|0.0091**	2.3 (1.2-4.3)	Overall
7-genes signature	cutp: 0.344|0.676|0.572	83|62	0.2|0.2	1.3 (0.88-1.9)	Event-Free
2-genes signature	median: 0.369	72|73	0.017|0.019*	1.8 (1.1-2.9)	Overall
2-genes signature	median: 0.369	72|73	0.00094|0.0011**	1.9 (1.3-2.9)	Event-Free
7-genes signature	median: -0.00186	73|72	0.68|0.68	1.1 (0.69-1.8)	Overall
7-genes signature	median: -0.00186	73|72	0.74|0.74	1.1 (0.72-1.6)	Event-Free

The 2-genes signature is comprised of *FAM101B* and *WDFY4.* The 7-genes signature is comprised of *CLEC11A, PRAME, AZU1, NREP, ARMH1, C1QBP, TRH* genes. The one-sided log-rank and Wald test significance are indicated as *P<.05, ** P<.01, ***P<.001. The number of patients are specified under "defined groups #Low I #High".

Supplementary Table 9: Survival analysis of differentially expressed genes in relapse- and CCR-associated dominant AML blasts.

Gene	Grouping method: cutpoint (cut-point | p value | percentile)	Defined groups #Low|#High	Log Rank p-value | HR p-value	Hazard Ratio, HR (95% CI)	Survival analysis
RFLNB/FAM101B	cutp: 5.61|0.0156*|0.49	71|74	0.00255|0.0032**	2.12 (1.29-3.48)	Overall
FLNA	cutp: 5.85|0.0582|0.448	65|80	0.0148|0.0164*	1.84 (1.12-3.03)	Overall
TRH	cutp: 3.45|0.00239**|0.566	82|63	0.000396|0.000626***	0.396 (0.233-0.674)	Overall
MPO	cutp: 8.16|0.00494**|0.448	65|80	0.00101|0.00135**	0.455 (0.281-0.736)	Overall
RFLNB/FAM101B	cutp: 5.19|0.0139*|0.372	54|91	0.00334|0.00387**	1.88 (1.22-2.88)	Event-Free
FLNA	cutp: 5.21|0.389|0.193	28|117	0.076|0.0787	1.6 (0.948-2.69)	Event-Free
TRH	cutp: 4.04|0.153|0.621	90|55	0.0252|0.0266*	0.626 (0.413-0.947)	Event-Free
MPO	cutp: 7.24|0.3|0.345	50|95	0.037|0.0384*	0.657 (0.442-0.978)	Event-Free
RFLNB/FAM101B	median: 5.61	71|74	0.00255|0.0032**	2.12 (1.29-3.48)	Overall
FLNA	median: 6.04	73|72	0.0296|0.0315*	1.7 (1.05-2.74)	Overall
TRH	median: 2.75	73|72	0.00436|0.00521**	0.495 (0.302-0.811)	Overall
MPO	median: 8.85	73|72	0.00515|0.00607**	0.504 (0.308-0.822)	Overall
RFLNB/FAM101B	median: 5.61	71|74	0.0107|0.0116*	1.66 (1.12-2.45)	Event-Free
FLNA	median: 6.04	73|72	0.449|0.449	1.16 (0.789-1.71)	Event-Free
TRH	median: 2.75	73|72	0.113|0.114	0.732 (0.496-1.08)	Event-Free
MPO	median: 8.85	73|72	0.335|0.335	0.827 (0.562-1.22)	Event-Free

Select genes, namely *RFLNB/FAM101B* and *FLNA* highly expressed in relapse-associated samples and *MPO* and *TRH* highly expressed in CCR-associated samples were assessed for association with OS and EFS using cutp and median based cut-point grouping methods in Survival genie tool[4]. The one-sided log-rank and Wald test significance are indicated as *P<.05, ** P<.01, *** P<.001. The number of patients are specified under "defined groups #Low I #High".

Supplementary Table 10: Survival analysis of differentially expressed genes in treatment resistant and treatment responsive cells clusters.

Gene	Grouping method: cutpoint (cut-point | p value | percentile)	Defined groups #Low |#High	Log Rank p-value | HR p-value	Hazard Ratio, HR (95% CI)	Survival analysis
CRHBP	cutp: 1.03|0.585|0.793	115|30	0.0555|0.0582	1.67 (0.982-2.84)	Overall
HOPX	cutp: 0.198|0.271|0.186	27|118	0.00644|0.00771**	0.479 (0.279-0.823)	Overall
TPM1	cutp: 0.416|0.00398*|0.483	70|75	0.00122|0.00165**	2.24 (1.36-3.71)	Overall
SEPP1	cutp: 0.34|0.0037**|0.549	80|65	0.000459|0.000667***	2.33 (1.43-3.79)	Overall
MSLN	cutp: 0.333|0.0155*|0.329	49|96	0.00168|0.00214**	0.473 (0.293-0.763)	Overall
KCNE5	cutp: 0.348|0.0389*|0.386	56|89	0.00792|0.009**	0.53 (0.329-0.853)	Overall
C1orf110	cutp: 0.00451|0.0882|0.383	108|37	0.00609|0.00715**	2 (1.21-3.3)	Overall
CRHBP	cutp: 0.413|0.0561|0.421	61|84	0.00866|0.00947**	1.71 (1.14-2.58)	Event-Free
HOPX	cutp: 0.615|0.0265*|0.448	65|80	0.00381|0.00431**	1.8 (1.2-2.7)	Event-Free
TPM1	cutp: 0.235|0.0565|0.255	37|108	0.00517|0.00609**	1.98 (1.22-3.24)	Event-Free
SEPP1	cutp: 0.101|0.0524|0.312	46|99	0.00948|0.0105*	1.78 (1.14-2.76)	Event-Free
C1orf110	cutp: 0.0042|0.0451*|0.3	103|42	0.000861|0.00107**	1.99 (1.32-3)	Event-Free
MSLN	cutp: 4.91|0.845|0.734	107|38	0.292|0.293	0.783 (0.496-1.24)	Event-Free
KCNE5	cutp: 0.531|0.221|0.441	64|81	0.0352|0.0365*	0.662 (0.449-0.974)	Event-Free
CRHBP	median: 0.568	73|72	0.998|0.998	1 (0.622-1.61)	Overall
HOPX	median: 0.919	73|72	0.985|0.985	1 (0.624-1.62)	Overall
TPM1	median: 0.449	73|72	0.00325|0.00397**	2.07 (1.26-3.38)	Overall
SEPP1	median: 0.215	72|73	0.00465|0.00554**	2.01 (1.23-3.29)	Overall
C1orf110	median: 0	86|59	0.0423|0.0444*	1.63 (1.01-2.62)	Overall
MSLN	median: 1.31	73|72	0.0406|0.0427*	0.605 (0.372-0.984)	Overall
KCNE5	median: 1.01	72|73	0.073|0.0754	0.645 (0.398-1.05)	Overall
CRHBP	median: 0.568	73|72	0.0379|0.0392*	1.51 (1.02-2.23)	Event-Free
HOPX	median: 0.919	73|72	0.0302|0.0314*	1.54 (1.04-2.28)	Event-Free
TPM1	median: 0.449	73|72	0.0991|0.101	1.38 (0.939-2.04)	Event-Free
SEPP1	median: 0.215	72|73	0.0329|0.0342*	1.52 (1.03-2.25)	Event-Free
C1orf110	median: 0	86|59	0.0106|0.0115*	1.65 (1.12-2.44)	Event-Free
MSLN	median: 1.31	73|72	0.307|0.308	0.818 (0.555-1.2)	Event-Free
KCNE5	median: 1.01	72|73	0.0507|0.0521	0.68 (0.461-1)	Event-Free

*The one-sided log-rank and Wald test significance is indicated as **P*<.05, ** *P*<.01, *** *P*<.001. The number of patients is specified under “defined groups #Low I #High”.

Supplementary Table 11: Significant pathways altered in therapy resistant vs. responsive blast cells.

Pathway	Resistant mean	Responsive mean	P value
WP_MAMMARY_GLAND_DEVELOPMENT_PATHWAY_PUBERTY_STAGE_2_OF_4	1.076	-0.068	5.71E-45
REACTOME_NR1H2_NR1H3_REGULATE_GENE_EXPRESSION_LINKED_TO_GLUCONEOGENESIS	1.071	-0.068	4.18E-21
WP_APOPTOSISRELATED_NETWORK_DUE_TO_ALTERED_NOTCH3_IN_OVARIAN_CANCER	1.028	-0.065	2.95E-47
REACTOME_MUSCLE_CONTRACTION	0.910	-0.058	3.86E-35
BIOCARTA_SLRP_PATHWAY	0.904	-0.057	5.34E-09
HALLMARK_ESTROGEN_RESPONSE_LATE	0.857	-0.054	3.49E-30
WP_PROSTAGLANDIN_SYNTHESIS_AND_REGULATION	0.815	-0.052	1.79E-33
HALLMARK_EPITHELIAL_MESENCHYMAL_TRANSITION	0.792	-0.050	3.15E-05
HALLMARK_UV_RESPONSE_DN	0.789	-0.050	2.94E-16
PID_INTEGRIN_A4B1_PATHWAY	0.789	-0.050	9.62E-26
REACTOME_SMOOTH_MUSCLE_CONTRACTION	0.788	-0.050	4.91E-29
REACTOME_DEFECTIVE_CHST6_CAUSES_MCDC1	0.764	-0.048	2.82E-08
WP_SPINAL_CORD_INJURY	0.759	-0.048	1.59E-30
REACTOME_DERMATAN_SULFATE_BIOSYNTHESIS	0.758	-0.048	0.001534
REACTOME_INTERLEUKIN_4_AND_INTERLEUKIN_13_SIGNALING	0.720	-0.046	1.75E-23
WP_FATTY_ACID_OMEGA_OXIDATION	0.711	-0.045	0.000132
HALLMARK_ANGIOGENESIS	0.702	-0.044	9.44E-11
NABA_PROTEOGLYCANS	0.686	-0.043	4.40E-05
WP_SELENIUM_MICRONUTRIENT_NETWORK	0.682	-0.043	2.88E-22
REACTOME_ECM_PROTEOGLYCANS	0.682	-0.043	0.032684
REACTOME_FORMYL_PEPTIDE_RECEPTORS_BIND_FORMYL_PEPTIDES_AND_MANY_OTHER_LIGANDS	0.681	-0.043	3.47E-25
PID_HNF3A_PATHWAY	0.664	-0.042	1.26E-22
REACTOME_STRIATED_MUSCLE_CONTRACTION	0.662	-0.042	1.74E-24
REACTOME_PRESYNAPTIC_FUNCTION_OF_KAINATE_RECEPTORS	0.650	-0.041	1.51E-22
BIOCARTA_PPARA_PATHWAY	0.649	-0.041	5.71E-21
REACTOME_ESR_MEDIATED_SIGNALING	0.647	-0.041	7.41E-24
REACTOME_SIGNALING_BY_NUCLEAR_RECEPTORS	0.643	-0.041	2.17E-22
REACTOME_CIRCADIAN_CLOCK	0.639	-0.040	5.66E-23
WP_NOCGMPPKG_MEDIATED_NEUROPROTECTION	0.638	-0.040	2.22E-22
HALLMARK_TGF_BETA_SIGNALING	0.634	-0.040	1.28E-16
PID_TCR_CALCIUM_PATHWAY	0.630	-0.040	2.64E-21
HALLMARK_MYOGENESIS	0.628	-0.040	1.30E-14
REACTOME_REGULATION_OF_INSULIN_SECRETION	0.626	-0.040	1.57E-18
BIOCARTA_AHSP_PATHWAY	0.622	-0.039	1.92E-15

Table shows top pathways (mean ssGSEA score>0.6, *P*<.001) in residual blasts cells in EOI samples. Significance was calculated using "linear.model" test in getSignificance function in escape package[7] and is specified for each pathway in the "*P* value" column in above table. Dx: n=4,173 cells from n=4 biologically independent samples, EOI: n=264 cells from n=5 biologically independent samples,

Supplementary Table 12: Survival analysis based on pathways enriched in EOI residual blasts.

Pathway	number of genes	Grouping method: cutpoint	Defined groups #Low|#High	Log Rank p-value | HR p-value	Hazard Ratio, HR (95% CI)	survival
REACTOME_MUSCLE_CONTRACTION	205	median, -0.708	72|73	0.00928**|0.0105*	1.9 (1.2-3.1)	overall
WP_FATTY_ACID_OMEGA_OXIDATION	15	median, -0.357	72|73	0.0172*|0.0188*	1.8 (1.1-2.9)	overall
NABA_PROTEOGLYCANS	35	median, -0.457	72|73	0.0162*|0.0178*	1.8 (1.1-3)	overall
WP_SELENIUM_MICRONUTRIENT_NETWORK	91	median, 0.276	72|73	0.0229*|0.0247*	1.7 (1.1-2.8)	overall
BIOCARTA_PPARA_PATHWAY	52	median, 0.361	72|73	0.00193**|0.00246**	2.1 (1.3-3.5)	overall
HALLMARK_MYOGENESIS	200	median, 0.586	72|73	0.00251**|0.00313**	2.1 (1.3-3.4)	overall
REACTOME_ESR_MEDIATED_SIGNALING	221	median, 0.649	72|73	0.00101**|0.00136**	2.2 (1.4-3.7)	overall
REACTOME_SIGNALING_BY_NUCLEAR_RECEPTORS	297	median, 0.788	72|73	0.00875**|0.00994**	1.9 (1.2-3.1)	overall
WP_NOCGMPPKG_MEDIATED_NEUROPROTECTION	48	median, -0.0219	73|72	0.00928**|0.0105*	1.8 (1.1-2.9)	overall
REACTOME_STRIATED_MUSCLE_CONTRACTION	36	median, -0.245	72|73	0.00928**|0.0105*	1.8 (1.1-2.9)	overall
HALLMARK_UV_RESPONSE_DN	144	median, 0.518	72|73	0.00531**|0.00589**	1.7 (1.2-2.6)	overall
REACTOME_MUSCLE_CONTRACTION	205	median, -0.708	72|73	0.0244*|0.0256*	1.6 (1.1-2.3)	event free
WP_FATTY_ACID_OMEGA_OXIDATION	15	median, -0.357	72|73	0.000479***|0.000612***	1.7 (1-2.7)	event free
NABA_PROTEOGLYCANS	35	median, -0.457	72|73	0.0814|0.0829	1.4 (0.96-2.1)	event free
WP_SELENIUM_MICRONUTRIENT_NETWORK	91	median, 0.276	72|73	0.0249*|0.0238*	1.6 (1.1-2.3)	event free
BIOCARTA_PPARA_PATHWAY	52	median, 0.361	72|73	0.00495**|0.00551**	1.7 (1.2-2.6)	event free
HALLMARK_MYOGENESIS	200	median, 0.586	72|73	0.0062**|0.00683**	1.7 (1.2-2.5)	event free
REACTOME_ESR_MEDIATED_SIGNALING	221	median, 0.649	72|73	0.0139*|0.0148*	1.6 (1.1-2.4)	event free
REACTOME_SIGNALING_BY_NUCLEAR_RECEPTORS	297	median, 0.788	72|73	0.0362*|0.0375*	1.5 (1.1-2.2)	event free
WP_NOCGMPPKG_MEDIATED_NEUROPROTECTION	48	median, -0.0219	73|72	0.0757|0.0757	1.4 (0.96-2.1)	event free
REACTOME_STRIATED_MUSCLE_CONTRACTION	36	median, -0.245	72|73	0.154|0.155	1.3 (0.9-2)	event free
HALLMARK_UV_RESPONSE_DN	144	median, 0.518	72|73	0.00751**|0.00648**	2 (1.2-3.2)	event free

Table shows overall survival and event free survival of selected pathways, upregulated in EOI residual blasts, which showed significant association with survival in TARGET AML data. The number of samples are specified under “defined groups #Low/high”. The log-rank and Wald test significance are indicated as *P<.05, ** P<.01, *** P<.001. The number of patients is specified under “defined groups #Low I #High”.

Supplementary References

1. Aran, D. *et al.* Reference-based analysis of lung single-cell sequencing reveals a transitional profibrotic macrophage. *Nat Immunol* **20**, 163-172 (2019).
2. Bailur, J.K. *et al.* Risk-associated alterations in marrow T cells in pediatric leukemia. *JCI Insight* **5**(2020).
3. Tickle, T.I.G., C.; Brown, M.; Haas, B. inferCNV of the Trinity CTAT Project. (2019).
4. Dwivedi, B., Mumme, H., Satpathy, S., Bhasin, S.S. & Bhasin, M. Survival Genie, a web platform for survival analysis across pediatric and adult cancers. *Sci Rep* **12**, 3069 (2022).
5. Szklarczyk, D. *et al.* STRING v11: protein-protein association networks with increased coverage, supporting functional discovery in genome-wide experimental datasets. *Nucleic Acids Res* **47**, D607-D613 (2019).
6. Fornerod, M. *et al.* Integrative Genomic Analysis of Pediatric Myeloid-Related Acute Leukemias Identifies Novel Subtypes and Prognostic Indicators. *Blood Cancer Discov* **2**, 586-599 (2021).
7. Borcherding, N. & Andrews, J. *escape: Easy single cell analysis platform for enrichment*. R package version 1.6.0. edn (2022).
