# Supplementary material for: Single-cell analysis reveals altered tumor microenvironments of relapse- and remission-associated pediatric acute myeloid leukemia
Source: Nat Commun. 2023 Oct 5;14:6209. doi: 10.1038/s41467-023-41994-0 (PMC10556066; doi:10.1038/s41467-023-41994-0)
Supplement: Supplementary file 3 — Reporting Summary [file 41467_2023_41994_MOESM3_ESM.pdf]

nature portfolio

Corresponding author(s): Manoj Bhasin

Last updated by author(s): Sep 22, 2023

Reporting Summary

Nature Portfolio wishes to improve the reproducibility of the work that we publish. This form provides structure for consistency and transparency in reporting. For further information on Nature Portfolio policies, see our Editorial Policies and the Editorial Policy Checklist.

Statistics

For all statistical analyses, confirm that the following items are present in the figure legend, table legend, main text, or Methods section.

n/a	Confirmed	
☐	☒	The exact sample size (n) for each experimental group/condition, given as a discrete number and unit of measurement
☐	☒	A statement on whether measurements were taken from distinct samples or whether the same sample was measured repeatedly
☐	☒	The statistical test(s) used AND whether they are one- or two-sided<br>*Only common tests should be described solely by name; describe more complex techniques in the Methods section.*
☒	☐	A description of all covariates tested
☐	☒	A description of any assumptions or corrections, such as tests of normality and adjustment for multiple comparisons
☐	☒	A full description of the statistical parameters including central tendency (e.g. means) or other basic estimates (e.g. regression coefficient) AND variation (e.g. standard deviation) or associated estimates of uncertainty (e.g. confidence intervals)
☐	☒	For null hypothesis testing, the test statistic (e.g. F, t, r) with confidence intervals, effect sizes, degrees of freedom and P value noted<br>*Give P values as exact values whenever suitable.*
☒	☐	For Bayesian analysis, information on the choice of priors and Markov chain Monte Carlo settings
☒	☐	For hierarchical and complex designs, identification of the appropriate level for tests and full reporting of outcomes
☐	☒	Estimates of effect sizes (e.g. Cohen's d, Pearson's r), indicating how they were calculated

Our web collection on statistics for biologists contains articles on many of the points above.

Software and code

Policy information about availability of computer code

Data collection	Chromium single cell sequencing technology from 10X Genomics was used for library construction following the manufacturer's guidelines. Sequencing was performed with the Illumina Novaseq (6000) S4 platform.
Data analysis	Raw scRNA-seq data was demultiplexed, aligned to the reference human genome (Hg38), and processed for single cell gene counting using Cell Ranger (v3.0.2 10X Genomics). The single cell count data was normalized using the SCTransform algorithm in Seurat v3.0. Bulk RNA-seq data was downloaded from TARGET (GDC portal) or publicly available studies (Fornerod et. al.) and normalized expression was calculated using the voom algorithm or by calculating the log(FPKM+1) values. Pathways and systems biology analysis was performed using the Ingenuity Pathway Analysis software package (IPA 9.0). Single-Sample GSEA (gene set enrichment analysis) was performed using molecular signature database v7.5 via the escape R package (Bioconductor). InferCNV is used to explore tumor single cell RNA-Seq data to identify evidence for large-scale chromosomal copy number variations, such as gains or deletions of entire chromosomes or large segments of chromosomes.

For manuscripts utilizing custom algorithms or software that are central to the research but not yet described in published literature, software must be made available to editors and reviewers. We strongly encourage code deposition in a community repository (e.g. GitHub). See the Nature Portfolio guidelines for submitting code & software for further information.

Data

Policy information about availability of data

All manuscripts must include a data availability statement. This statement should provide the following information, where applicable:

- Accession codes, unique identifiers, or web links for publicly available datasets
- A description of any restrictions on data availability
- For clinical datasets or third party data, please ensure that the statement adheres to our policy

The raw scRNA-seq data generated in this study have been deposited in the Gene Expression Omnibus database under accession code GSE235923 [https://www.ncbi.nlm.nih.gov/geo/query/acc.cgi?acc=GSE235923]. Due to IRB guidelines and patient/legal consent constraints, raw sequencing files in the FASTQ format are not publicly shared. However, this data can be provided upon request, subject to appropriate IRB approval and data transfer agreement. An interactive data resource and analytical tool developed based on this AML single-cell data is available online at https://bhasinlab.bmi.emory.edu/PediatricSC/. Publicly available datasets utilized in this study are available via the National Cancer Institute GDC Portal (https://portal.gdc.cancer.gov/projects/TARGET-AML) and the St. Jude Genomics Platform (https://platform.stjude.cloud/data/publications).

Human research participants

Policy information about studies involving human research participants and Sex and Gender in Research.

Reporting on sex and gender	We did not provide sex and gender as we did not consider these factors in our analysis.
Population characteristics	Patient ages range from 1.5 to 17.8 years. All patient ages are located in supplementary table 1.
Recruitment	Samples were obtained as part of a precision medicine study of Aflac Cancer and Blood disorder center from Children's Healthcare of Atlanta Pediatric Biorepository that was approved by the Emory institutional review board. Patients provided written informed consent that permitted the use of biological material in accordance with a protocol that was approved by the institutional review board. BM core biopsies were obtained at the initial presentation of AML (Dx), at the time of therapy remission; post-therapy (EOI), and at the time of relapse (Rel) as part of routine hematopathology evaluation.
Ethics oversight	Samples were obtained as part of a precision medicine study of Aflac Cancer and Blood disorder center from Children's Healthcare of Atlanta Pediatric Biorepository that was approved by the Emory institutional review board.

Note that full information on the approval of the study protocol must also be provided in the manuscript.

Field-specific reporting

Please select the one below that is the best fit for your research. If you are not sure, read the appropriate sections before making your selection.

☒ Life sciences ☐ Behavioural & social sciences ☐ Ecological, evolutionary & environmental sciences

For a reference copy of the document with all sections, see nature.com/documents/nr-reporting-summary-flat.pdf

Life sciences study design

All studies must disclose on these points even when the disclosure is negative.

Sample size	Sample sizes were not predetermined based on statistical methods, but were selected according to the standards in the field (at least three biological replicates per clinical group). Additional validation data were produced and obtained, increasing the number of biologically independent samples. Sample size was deemed sufficient based on the detection of expected cell types in bone marrow.
Data exclusions	The quality filtering on scRNA-seq data was performed by multiple filtering parameters including filtering out cells with >25% of mitochondrial genes and lower genes expression capture (< 200 genes), and genes only uniquely expressed in < 3 cells in the dataset.
Replication	Most of biological findings in study are based on expression profile based on >50 biologically independent cells, therefore have minimal chances of being false positives. Additionally we have also replicated our findings through independent validation by generating new data as well as using publicly available data.
Randomization	AML patients were divided into patients with Clinical Remission and relapse.
Blinding	Blinding was not required in this study and investigators were not blinded to group allocation as all data in this study was analyzed following the same methodology within defined experimental groups.

Reporting for specific materials, systems and methods

We require information from authors about some types of materials, experimental systems and methods used in many studies. Here, indicate whether each material, system or method listed is relevant to your study. If you are not sure if a list item applies to your research, read the appropriate section before selecting a response.

Materials & experimental systems

n/a	Involved in the study
☒	☐ Antibodies
☒	☐ Eukaryotic cell lines
☒	☐ Palaeontology and archaeology
☒	☐ Animals and other organisms
☒	☐ Clinical data
☒	☐ Dual use research of concern

Methods

n/a	Involved in the study
☒	☐ ChIP-seq
☒	☐ Flow cytometry
☒	☐ MRI-based neuroimaging
